# Modeling the Ocular Pharmacokinetics and Pharmacodynamics of Ranibizumab for Improved Understanding and Data Collection Strategies in Ocular Diseases

**DOI:** 10.1167/iovs.66.6.20

**Published:** 2025-06-06

**Authors:** Jessica R. Crawshaw, Eamonn A. Gaffney, Michael Gertz, Philip K. Maini, Antonello Caruso

**Affiliations:** 1 Wolfson Centre for Mathematical Biology, Mathematical Institute, University of Oxford, Oxford, United Kingdom; 2 Roche Pharmaceutical Research and Early Development, Pharmaceutical Sciences, Roche Innovation Center Basel, Switzerland

**Keywords:** pharmacokinetics, pharmacodynamics, Bayesian inference, ranibizumab, mathematical ophthalmology

## Abstract

**Purpose:**

Improving our understanding of the ocular pharmacokinetics and pharmacodynamics of anti-vascular endothelial growth factor (VEGF) therapies, such as ranibizumab, is essential to enhance treatment strategies for a range of retinal diseases, and will help inform the development of novel anti-VEGF drug candidates.

**Methods:**

In this study, we examine a two-compartment pharmacokinetic/pharmacodynamic model of an intravitreal ranibizumab injection to understand its impact on ocular VEGF suppression. We use Bayesian inference to infer the model parameters from aqueous humor data extracted from healthy cynomolgus macaques. We leverage this approach to explore various sources of uncertainty in the data, offering practical recommendations for minimizing avoidable uncertainty.

**Results:**

The model provides a robust description of ranibizumab pharmacokinetics and pharmacodynamics, identifying the recovery region of the aqueous humor VEGF concentration–time profile as critical for the precise estimation of parameters. Our results advocate focusing on this region in future studies for optimal data collection. We consider standard data correction techniques to reduce the data uncertainty introduced by the lower limit of quantification, identifying the most preferable technique for this model and data. Using a Bayesian approach we obtain an inferred mean posterior distribution of 1459 ± 98 pM for the ranibizumab dissociation constant, a pharmacodynamic parameter with notable variability across the literature.

**Conclusions:**

This study extends our understanding of the ocular pharmacokinetics and pharmacodynamics of ranibizumab and provides theoretical insights for enhanced data collection schemes to be considered for clinical trials and in the development of novel anti-VEGF therapies.

Intravitreal (IVT) administration of anti-vascular endothelial growth factor (VEGF) therapy is currently the preferred treatment strategy for vascular retinal diseases, such as neovascular AMD (wet AMD), diabetic retinopathy, and diabetic macular edema.^1^^–^^3^ As the population ages, the prevalence of wet AMD is projected to increase, reaffirming its status as the leading cause of central blindness in individuals aged 50 and older.^4^^–^^6^ Consequently, there is an increasingly urgent demand for enhanced treatment strategies and prevention measures. VEGF, a proangiogenic homodimeric molecule, serves a central role in neovascular ocular diseases, driving pathological vascular growth. To target ocular VEGF, specifically designed anti-VEGF macromolecules, such as ranibizumab (Lucentis, Genentech Inc., San Francisco, CA, USA^1^^,^^7^^,^^8^), and aflibercept (Eylea, Regeneron Pharmaceuticals, Tarrytown, NY, USA^2^^,^^9^^,^^10^), are delivered via IVT injections. By binding to VEGF, these anti-VEGF macromolecules block the interaction between VEGF and the VEGF receptors present on the surface of vascular endothelial cells within the retina and choroid,^11^ thereby impeding the proangiogenic functions of VEGF and slowing disease progression.

Ranibizumab is a recombinant humanized monoclonal antibody fragment targeting VEGF that has been shown to improve visual acuity in patients with wet AMD and is approved for the treatment of this condition.^8^^,^^12^ Along with other VEGF-targeting drugs, ranibizumab effectively manages wet AMD, diabetic retinopathy, and diabetic macular edema (among other retinal vascular diseases), slowing disease progression.^11^^,^^13^^–^^22^ However, these anti-VEGF therapies have a relatively short ocular half-life (approximately 1 week) owing to the rapid transport processes within the eye. Although it is generally accepted that treatment intervals of 1 to 3 months are required, the substantial variability in treatment response among individuals makes it challenging to rationally determine improved scheduling for repeated injections in each patient. This underscores the need to obtain a better understanding of the ocular pharmacokinetics and pharmacodynamics (PK/PD) of anti-VEGF drugs such as ranibizumab, especially the determinants of interpatient variability and measurement uncertainty, as well as a need to develop longer-acting treatments that can sustain therapeutic levels over extended periods.^23^ Moreover, improved PK/PD insights could offer optimal treatment schedules, thus reducing the number of treatments required while ensuring therapeutic drug levels are maintained for extended periods.

Our current understanding of ocular ranibizumab PK/PD is incomplete. There exists a significant disparity between the ranibizumab dissociation constants (*K*_*D*_) reported in the current literature.^24^^–^^40^ Moreover, the factors influencing ocular drug retention have yet to be fully classified and quantified. To quantitatively characterize the ocular PK/PD of ranibizumab, in this study we consider a two-compartment PK/PD model of ranibizumab^39^ in the context of time-series aqueous humor VEGF and ranibizumab concentration data obtained from healthy cynomolgus macaque eyes.^41^ PK/PD models are highly useful mathematical and computational tools in the field of pharmaceutical research and development because they provide a structured framework for understanding and quantifying the relationships between drug exposure and therapeutic outcomes. These models enhance efficiency in drug design and expedite the selection of therapeutic candidates. Additionally, PK/PD models enable researchers and clinicians to predict drug behavior in diverse patient populations, evaluate the impact of different dosing schedules, and develop tailored treatment strategies. As pharmaceutical research continues to evolve, PK/PD models are becoming a useful tool in understanding the complexities of drug actions, ultimately leading to more effective and customized treatments for various medical conditions.

To gain practical insights from PK/PD models, it is necessary to validate and calibrate these models using experimental data. To this end, Bayesian inference emerges as a useful tool for inferring parameter distributions from experimental data, distinguishing itself by its ability to accommodate non-normally distributed parameters, incorporate prior information, and handle and characterize the uncertainty in the data. Bayesian inference serves to map uncertainty in the data into uncertainty in parameter sets. Uncertainties in data arise from several sources, including measurement errors, constraints introduced by data collection schedules, limits of quantification, and the inherent noise within the physiological system. Predicting the most informative sampling schedule and data correction technique to accommodate such uncertainty is challenging. Indeed, there are no system-specific recommendations for data correction techniques and data collection scheduling. Nonetheless, strategically mitigating uncertainty from these sources reduces the uncertainty carried into parameter estimates. This paper examines strategies for minimising avoidable uncertainties by understanding the variability introduced by experimental choices, such as sampling schedules and data correction techniques. This nuanced approach aims to enhance the reliability of parameter estimates, producing a more robust and accurate description of the underlying physiological dynamics.

In this study, we quantitatively characterize the ocular PK/PD of ranibizumab in a nonclinical animal model (cynomolgus macaque). We analyze the time-series aqueous humor VEGF and ranibizumab data extracted from the Niwa et al.^41^ study using a Bayesian inference strategy with the semi-mechanistic two-compartment model of ocular transport processes first presented by Hutton-Smith et al.^39^ (Fig. 1). This analysis provides insight into the PK/PD properties of ranibizumab in the cynomolgus macaque eye. Using this model, we classify the most sensitive parameters influencing the ocular pharmacodynamic duration of ranibizumab. Building on this Bayesian approach, our second objective is to examine common data sampling strategies and data correction techniques to enhance evidence-based quantification of PK/PD for current and prospective anti-VEGF therapeutics administered by IVT injection.

**Figure 1.**
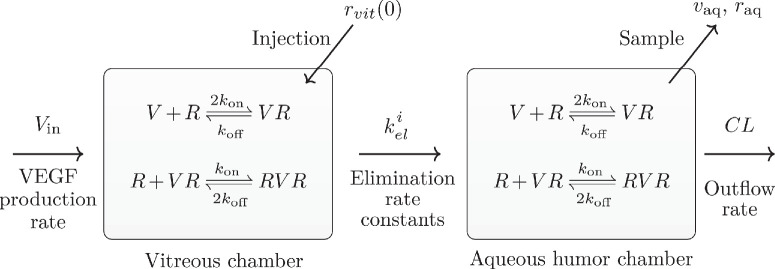
Schematic diagram of the two-compartment pharmacodynamic model of VEGF (V) and ranibizumab (R) interactions in the eye. We assume that the eye is composed of the vitreous (left) and the aqueous humor (right) compartments. Experimental samples of VEGF and ranibizumab were collected from the aqueous humor compartment, and the injection of ranibizumab at time *t* = 0 is delivered to the vitreous compartment. Figure adapted from Hutton-Smith et al.^39^

## Methods

To quantitatively characterize the intraocular PK/PD of ranibizumab in the cynomolgus macaque, we consider the well-mixed semi-mechanistic two-compartment model of ocular VEGF (V) suppression after a single IVT ranibizumab (R) treatment originally presented by Hutton-Smith et al.^39^ (Fig. 1). In this two-compartment model, a bolus of ranibizumab (0.5 mg) is delivered directly to the vitreous and is cleared to the aqueous humor through a first-order transfer process (Fig. 1). Following from previous work, we assume that the transfer of ranibizumab to the retina is small (∼10%) compared to the transfer to the aqueous humor, so the retinal compartment can be neglected. VEGF contains two identical binding sites for ranibizumab, facilitating the reversible formation of the VEGF-ranibizumab (VR) complex and the ranibizumab-VEGF-ranibizumab (RVR) complex. The mass action dynamics of these processes can be expressed as

$$\begin{array}{ccc} & & V+R\;\overset{2k_{\text{on}}}{\underset{k_{\text{off}}}{⇌}}\;VR,\\ & & R+VR\;\overset{k_{\text{on}}}{\underset{2k_{\text{off}}}{⇌}}\;RVR, \end{array}$$

where *k*_on_ is the association rate constant, *k*_off_ is the dissociation rate constant, and the dissociation constant is given by *K*_*D*_ = *k*_off_/*k*_on_. As shown in Figure 1, these reactions occur in both the vitreous and aqueous humor compartments, with unidirectional transfer of each species, *i*, from the vitreous to the aqueous humor compartment, described by the elimination rate constants $k_{\text{el}}^{i}$. From the aqueous humor, large molecules are then cleared into the circulation, predominantly via Schlemm’s canal, by the rapid process of aqueous humor turnover. For simplicity, we assume that the clearance rate, *CL*, and the retinal VEGF production rate, *V*_in_, are constant. We assume that the system is well-mixed^39^ and can therefore be described by the system of coupled non-linear ordinary differential equations (S1.1), presented in Section S1 of the Supplementary Material. Parameter notations, descriptions, and the currently accepted values from the literature for cynomolgus macaques are shown in Table 1.

**Table 1.** Model Parameter Values Sourced From the Current Literature With Standard Units. We Present the Range of Mean Values Taken From the Literature. The Parameters σ_1_ and σ_2_ Are not Defined in the Literature, but Are Presented in Figure 3 and Provide Important Information About the Errors in the Data

Parameter	Value	Units	Description	References
*k*_off_	0.85	day^−1^	Dissociation rate constant	24
*k*_on_	0.019 − 0.024	pM^−1^ · day^−1^	Association rate constant	24
*K*_D_	1.4 − 23,000	pM	Dissociation constant	24–40,42

*CL*	2.16 − 3.77	mL · day^−1^	Aqueous clearance rate constant	43–48
$V_{\text{vit}}$	1.8 − 2.3	mL	Vitreous volume	49–56
$V_{\text{aq}}$	0.101 − 0.109	mL	Aqueous humor volume	43,46,56–58
*V*_in_	unknown	pM·mL · day^−1^	VEGF production rate	–

$k_{el}^{r}$	0.300	day^−1^	VEGF elimination rate constant	
$k_{el}^{v}$	0.309	day^−1^	Ranibizumab elimination rate constant	Calculated based on 41 and 39
$k_{el}^{c}$	0.245	day^−1^	VR elimination rate constant	
$k_{el}^{h}$	0.210	day^−1^	RVR elimination rate constant	

σ_1_	Inferred	pM	Inferred error in the free VEGF data	
σ_2_	Inferred	pM	Inferred error in the free ranibizumab data	

## Model Analysis

To obtain estimates for each model parameter, we use Bayesian inference to analyze time-series pharmacokinetic data extracted from the aqueous humor of intact cynomolgus macaques following a single IVT injection of ranibizumab (0.5 mg/50 µL), as reported by Niwa et al.^41^ (figs. 1 and 3 therein). In doing so, we also consider several key sources of uncertainty inherent in the data, namely:

U1: The noise intrinsic to the biological system;
U2: The error introduced by limits of quantification;
U3: The noise introduced by the assumption that only free VEGF is measured; and
U4: The accuracy limitations introduced by the data collection schedule.

**Figure 2.**
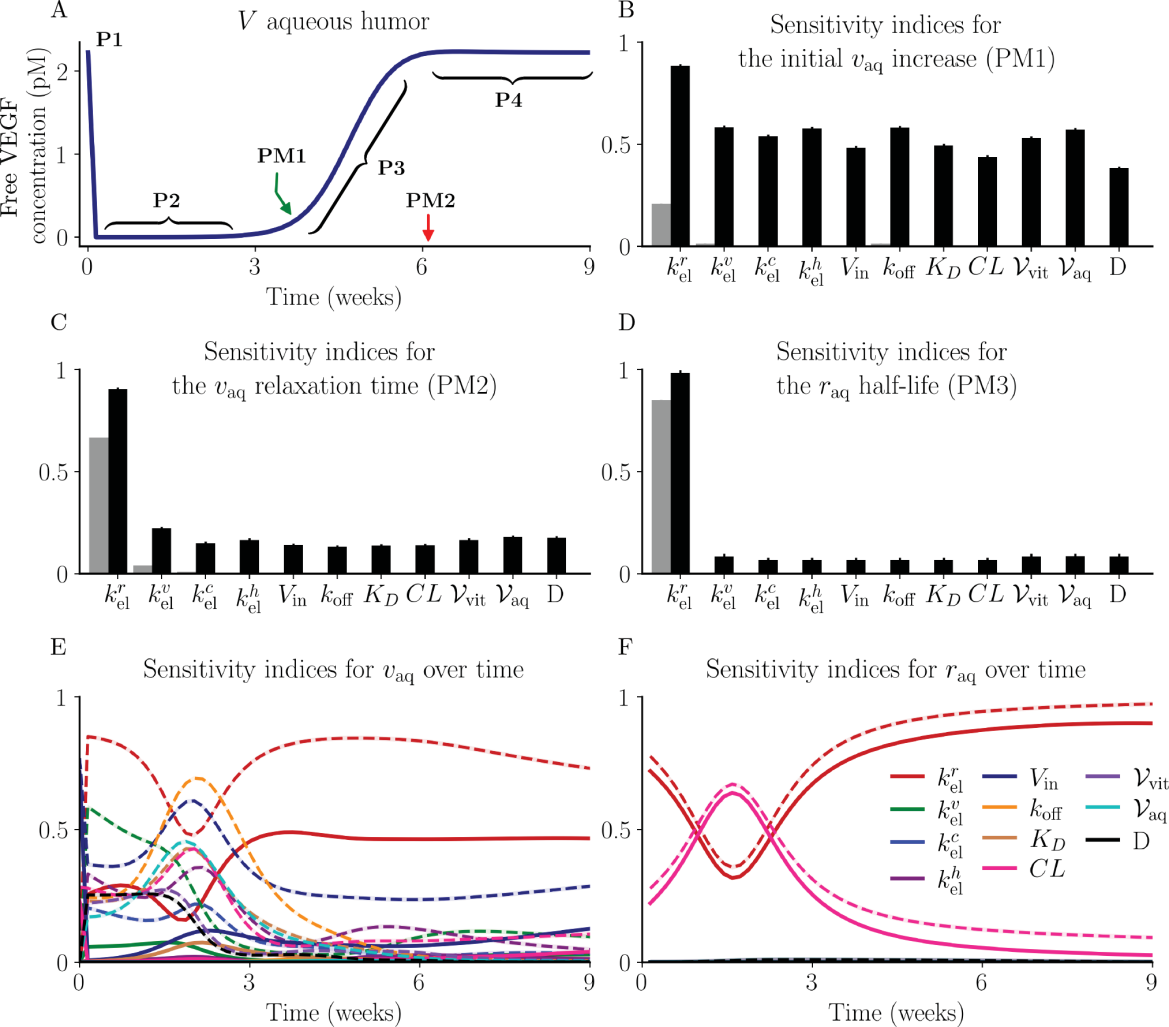
Sensitivity indices for each parameter considering the key pharmacokinetic metrics and *v*_aq_ and *r*_aq_ over time. (**A**) The key pharmacokinetic metrics (PM) and phases (P) annotated across a sample aqueous VEGF concentration profile: PM1 = time of initial VEGF increase; PM2 + VEGF relaxation time; P1 = the initial VEGF concentration data point; P2 = region of VEGF suppression; P3 = the VEGF transition region; and P4 = the VEGF return to equilibrium. (**B**–**D**) The first-order (*gray*) and total sensitivity indices (*black*) for (**B**) the point of initial *v*_aq_ return (PM1), (**C**) the *v*_aq_ relaxation time (PM2), and (**D**) the *r*_aq_ half-life (PM3). For each sensitivity index in **B** through **D**, the lines protruding from the bars give the upper region of the 95% CI. Note that some values are near zero and the first order sensitivity indices for many parameters are near zero. (**E** and **F**) The first-order (*solid*) and total sensitivity indices (*dashed*) change over time as *v*_aq_ and *r*_aq_ evolve. The 95% CI is shown in the narrow shaded region in **E** and **F**, which is often too small to be visible. The color code for **E** and **F** is shown in **F**.

**Figure 3.**
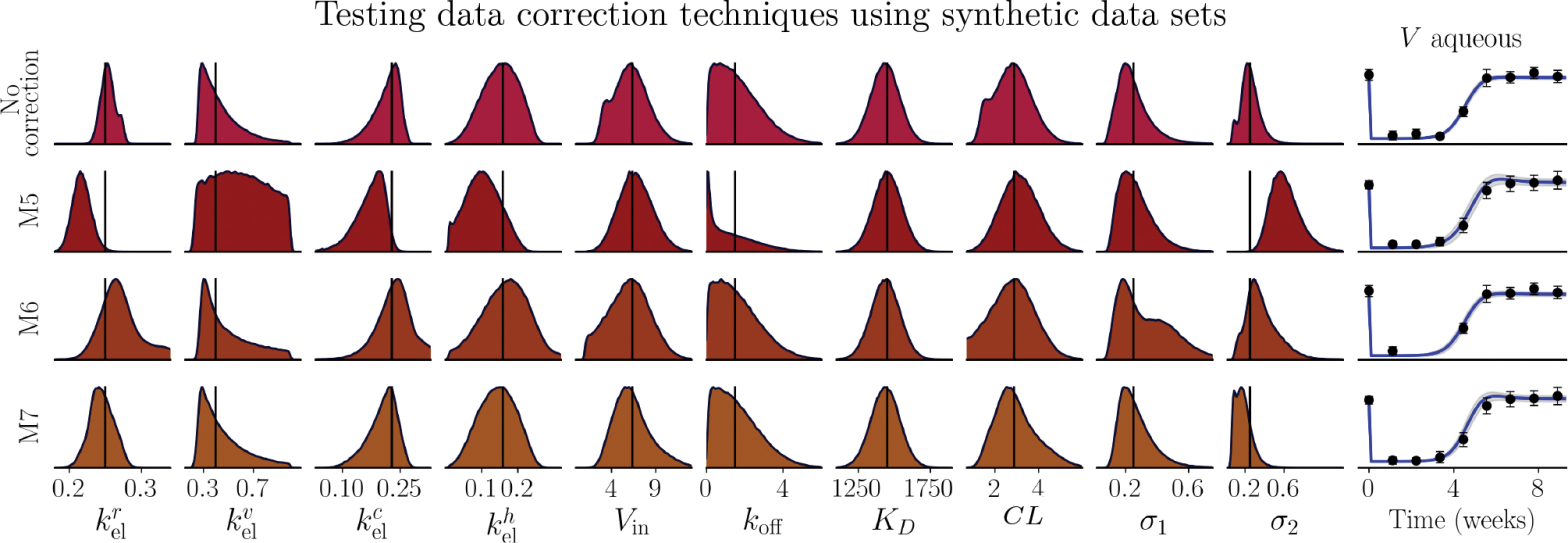
Inferred posterior distributions obtained from synthetic data without data correction (*top row*), and following data correction techniques M5 (*second row*), M6 (*third row*), and M7 (*fourth row*). Each distribution has been normalized in the plot by the maximum of the distribution. The model fits following each correction technique are shown in the rightmost column, and the reference parameter values used to generate the synthetic data are shown by the *black vertical lines*.

The noise in the data introduced by each source of uncertainty contributes to the uncertainty in the parameter estimates obtained using Bayesian inference. We quantify the impact of each source of uncertainty by examining several data correction techniques (to address U2), challenging the assumption that only free VEGF is measured by the enzyme-linked immunosorbent assay (ELISA) technique (to address U3) and considering the impact of different data collection schedules (to address U4). Understanding how to reduce the variation from U2 to U4 enables us to reduce the uncertainty in the parameter estimate from sources other than the noise intrinsic to the biological system (U1). We begin by identifying which parameters will be most influenced by uncertainty in the data using a global sensitivity analysis.

### The Ranibizumab Elimination Rate Constant Is the Paramount Model Parameter

We conducted a global sensitivity analysis using the extended Fourier amplitude sensitivity test (eFAST) to assess the sensitivity of the model output to uncertainties in the model parameters.^59^ eFAST is a variance-based method that decomposes the model output variance by means of spectral analysis to compute both the first-order (*S*_*i*_) and total-order sensitivity indices (*S*_*T*,*i*_) for a given parameter (*i*).^59^ The first-order sensitivity indices (*S*_*i*_) quantify the model output variance for variation in parameter *i* when all other parameters are fixed, and the total-order sensitivity indices (*S*_*T*,*i*_) quantify the variance due to parameter *i* and incorporating the interactions with other parameters.^60^ These indices identify which model parameters will have the greatest impact on model output. We considered the following three pharmacokinetic metrics (PM) as our model output:

PM1: The time of the initial uplift of aqueous humor VEGF following ranibizumab administration (see Fig. 2A);
PM2: The time to re-establish VEGF steady-state following ranibizumab administration (see Fig. 2A); and
PM3: The ranibizumab half-life.

The first two pharmacokinetic metrics, PM1 and PM2, are labeled on the sample aqueous humor VEGF concentration profile shown in Figure 2A, by the green and red arrows, respectively, along with the four distinct phases of the aqueous humor VEGF concentration profile. We considered the variance of each pharmacokinetic metric, as well as the variance in aqueous humor VEGF and ranibizumab concentrations at each time point over a 9-week period, with respect to the 10 model parameters and a dummy parameter (D). The dummy parameter was included to quantify the artefacts introduced by the eFAST algorithm. We implemented the eFAST method using the Python v3.9.1 software package SALib v.1.4.5^61^^,^^62^ (https://salib.readthedocs.io). Four search curves were employed, each consisting of 10,000 samples, with each curve randomly resampled 10,000 times, with the confidence interval set at 95%.^59^

Figures 2B–D show the first-order (*S*_*i*_, gray) and total-order sensitivity indices (*S*_*T*,*i*_, black) for each model parameter at PM1, PM2, and PM3, respectively. It is evident that $k_{\text{el}}^{r}$ is the most influential parameter for each pharmacokinetic metric, with both the first-order (gray) and total-order sensitivity indices (black) showing dominance in each pharmacokinetic metric. The sensitivity indices for all other parameters are less than 0.2 or are comparable with the dummy variable and therefore are considered to have a relatively insignificant influence on these key metrics. The sensitivity indices for aqueous humor VEGF and ranibizumab concentrations are functions of time, and are shown in Figures 2E and F, respectively, with the solid and dashed lines showing the first-order (*S*_*i*_) and the total-order sensitivity indices (*S*_*T*,*i*_), respectively. Again, we see that $k_{\text{el}}^{r}$ (red) is the most influential parameter. The total-order sensitivity indices for *k*_off_ (orange) and *V*_in_ (dark blue) peak briefly at early time (<25 days) and late time (>75 days), respectively, for *v*_aq_ (Fig. 2E). Initially (<25 days) *CL* (pink) has a notable influence on *r*_aq_ with a peak in the first-order and total-order sensitivity indices at 10 days. The sensitivity indices over time for the other species are shown in Supplementary Figure S3.1.

**Figure 4.**
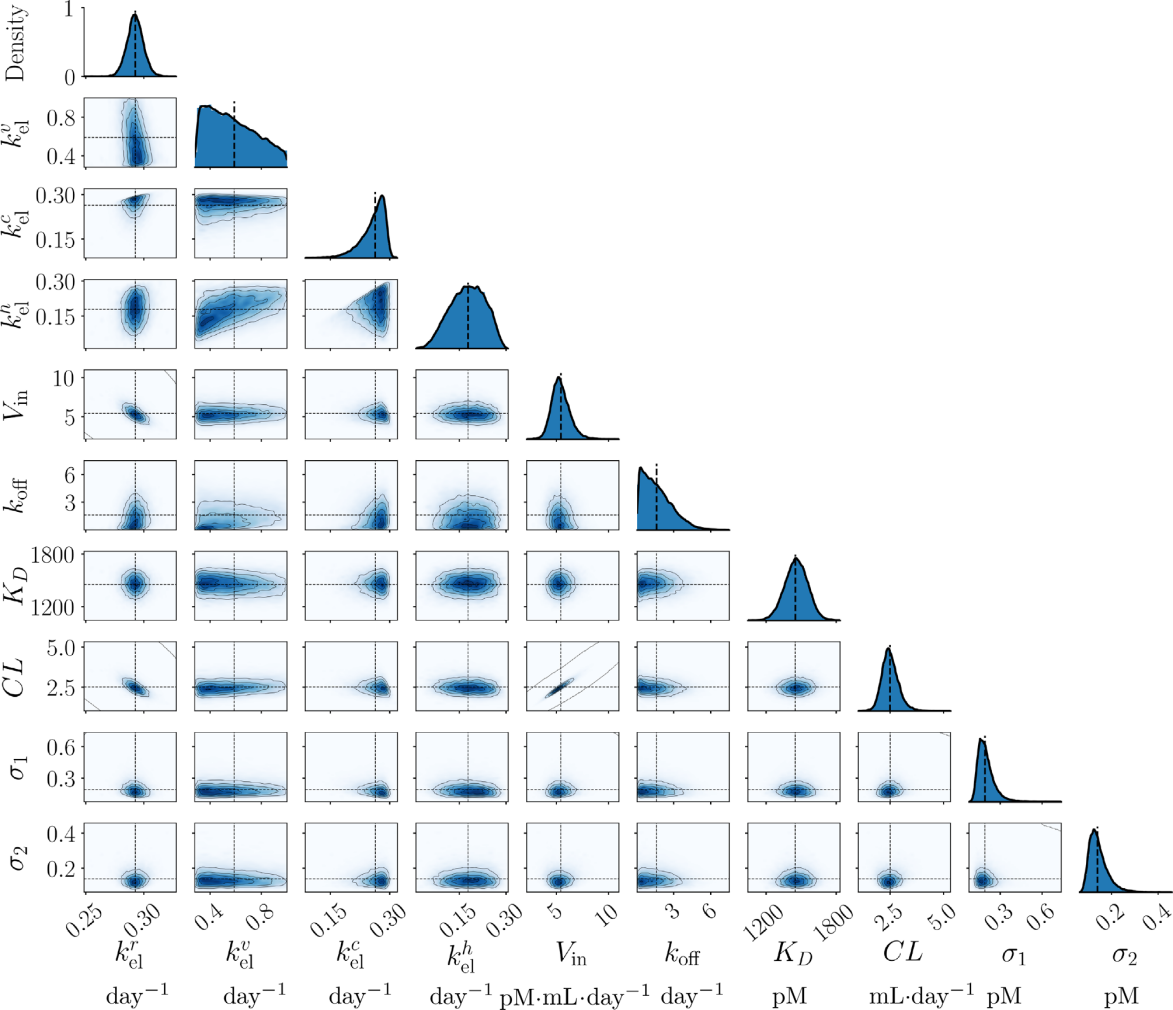
The posterior distributions inferred from the Niwa et al. study^41^ for each model parameter are shown along the diagonal subplots, while the off-diagonal subplots show the pairwise marginal posterior distributions.

**Figure 5.**
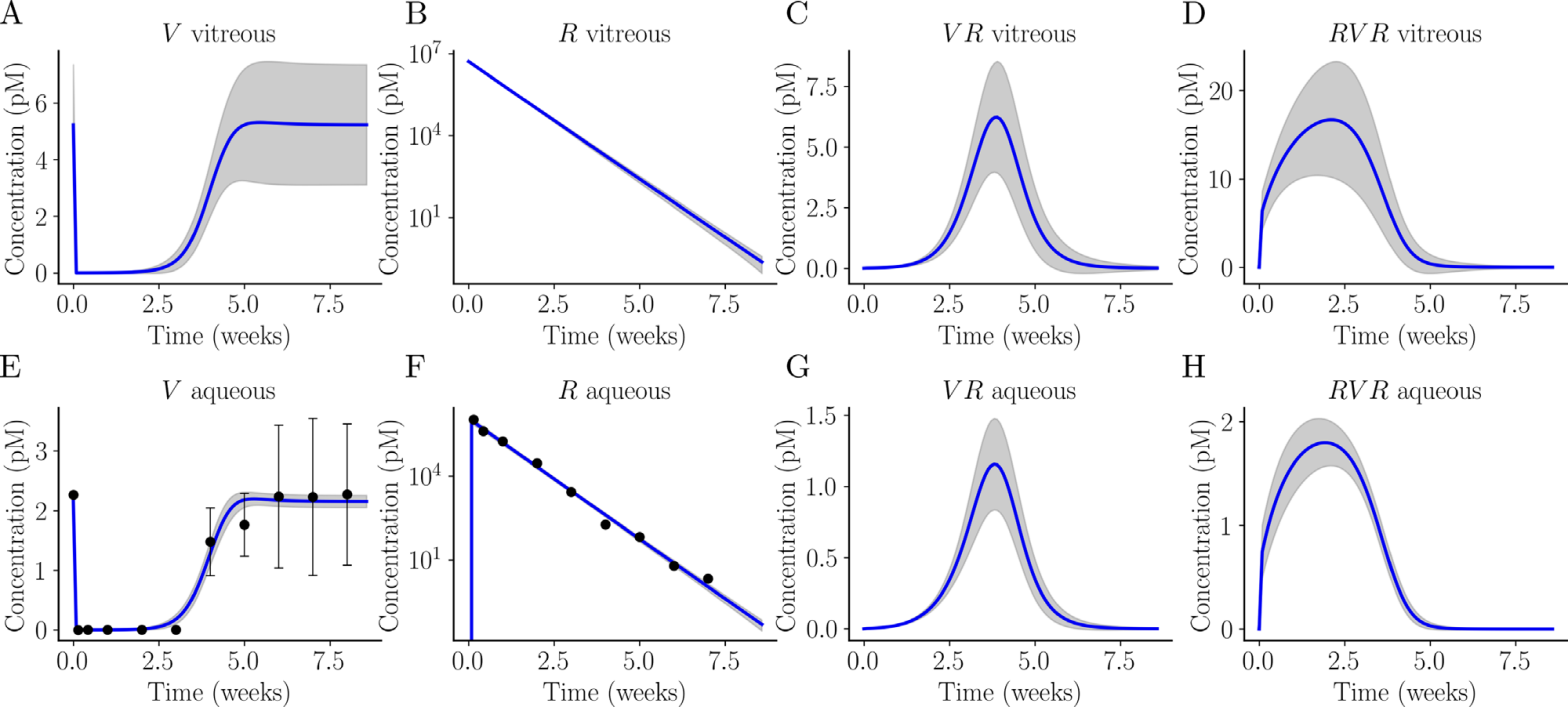
Semi-mechanistic two-compartment model of intraocular ranibizumab and VEGF PK/PD following a single intravitral bolus of ranibizumab. The mean model fits using data extracted from Niwa et al.^41^ are shown in *blue*. The *gray regions* show 1 SD around the mean model fit. From left to right, the top row shows the vitreous concentration of (**A**) VEGF (V), (**B**) ranibizumab (R), (**C**) partially bound VEGF (VR), and (**D**) fully bound VEGF (RVR). Similarly, the bottom row (**E**–**H**) shows the aqueous concentration of each molecular species in the same order. The mean SD of the aqueous VEGF and the aqueous ranibizumab data extracted from Niwa et al.^41^ are shown by the black circles and error bars in **E** and **F**.

**Figure 6.**
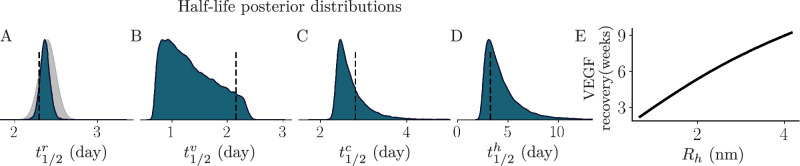
The posterior distributions for the molecular half-life values for (**A**) ranibizumab ($t_{1/2}^{r}$), (**B**) VEGF ($t_{1/2}^{v}$), (**C**) VR ($t_{1/2}^{c}$), and (**D**) RVR ($t_{1/2}^{h}$). The dashed line in (**A**) denotes the estimated ranibizumab half-life from Niwa et al.,^41^ and the dashed lines in (**B**–**D**) denote the estimated half-life calculated using the Hutton-Smith et al.^39^ scaling relationship. The gray normal distribution shows the distribution of ranibizumab half-life values sourced from the literature as presented by Caruso et al.^68^ (**E**) The theoretical relationship between the aqueous humor VEGF recovery time (weeks) with anti-VEGF antibody hydrodynamic radius (nm).

**Figure 7.**
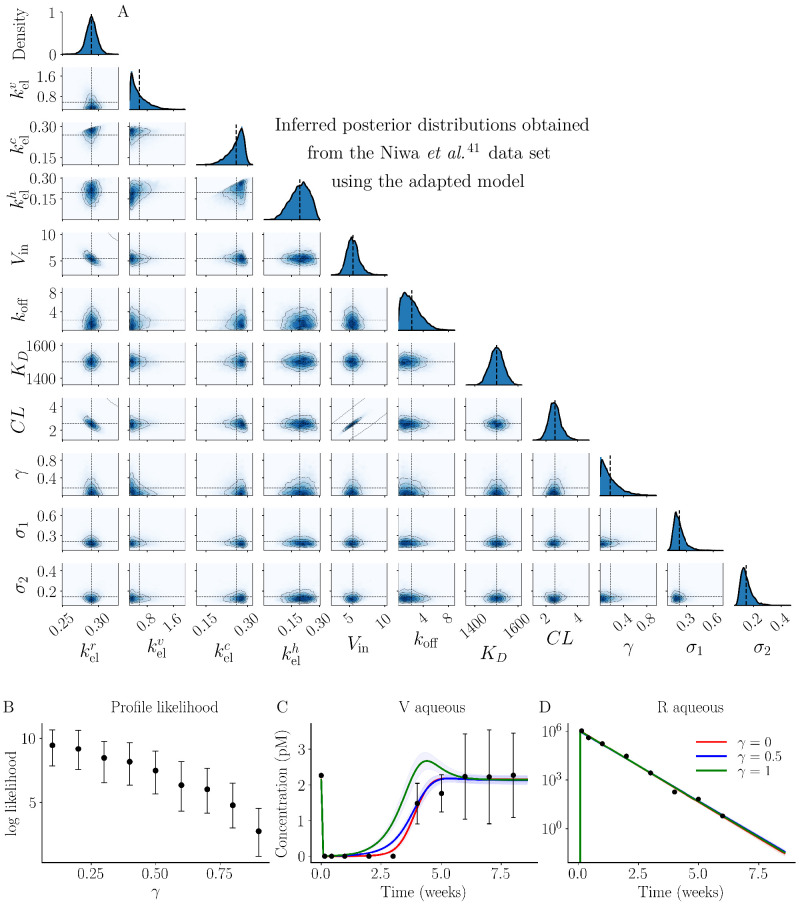
(**A**) The posterior distributions inferred from the Niwa et al.^41^ data set assuming the measured aqueous VEGF contains some proportion (γ) of the partially boundary VEGF (Equation 4). The *upper diagonal* panels show the marginal posterior distributions for each parameter, while the *off-diagonal panels* show the 2D marginal joint posterior distribution for each parameter pair. (**B**) This plot shows the mean and interquartile range of the log profile likelihood, which decreases as γ increases. Plots (**C**) and (**D**) show the optimal model fits for aqueous humor VEGF and ranibizumab, respectively, when γ is fixed at zero, 0.5, and 1.

**Figure 8.**
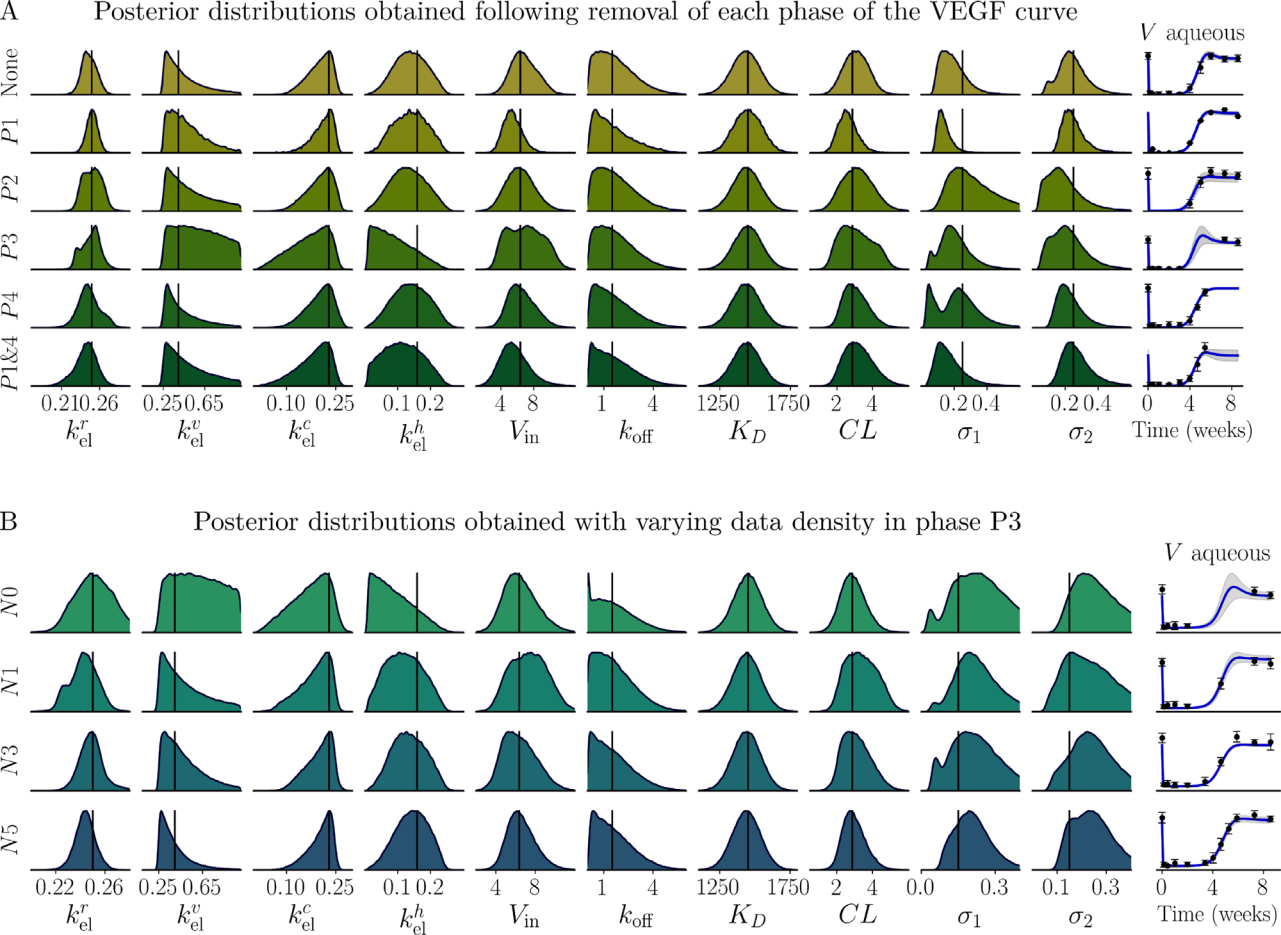
Inferred posterior distributions for each parameter obtained from ten series of synthetic data sets following the removal of a selected phase of the VEGF curve (**A**) or with varying data density in the transition region (P3) of the VEGF curve (**B**). (**A**) Inferred posterior distributions with full data sets (*row 1*) and then following the removal of P1, the initial data point (*row 2*); P2, the suppressed region (*row 3*); P3, the transition region (*row 4*); P4, the VEGF return to equilibrium (*row 5*); or the initial data point and the VEGF return to equilibrium (*row 6*). (**B**) The inferred posterior distributions become increasingly informative for the elimination rate constants and *V*_in_ as the number of data points in the VEGF transition region (P3) increases from zero data points (*top row*; N0), to one data point (*second row*; N1), to three data points (*third row*; N3), to finally five data points (*bottom row*; N5). The *right most column* shows the model fits for each synthetic data set in both (**A**) and (**B**). All distributions represent the mean distribution obtained from ten synthetic data sets and are normalized to the maximum value of the distribution.

### A Bayesian Approach Provides Novel Insight Into the PK/PD of Ranibizumab

Obtaining accurate information on model parameters from experimental data is a fundamental requirement when developing accurate and predictive models. Previous work studying the ocular PK/PD in humans and cynomolgus macaques have approached model parameter estimation using least-squares regression and nonlinear mixed effects.^39^^,^^40^ However, these approaches either neglect prior information available in the literature, often assume model parameters can be sampled from a normal distribution, or neglect the variability in the model parameters. As such, we have used Bayesian inference to infer the model parameter distributions from experimental data. Bayesian inference is a statistical method that updates the probability of a hypothesis as new data are obtained. This approach integrates prior knowledge, encapsulated in a prior distribution, with new experimental data to estimate the posterior distribution, which represents the updated probability distributions of the model parameters. This approach translates the uncertainty in the data into uncertainty in the model parameters. Based on the existing literature values for each parameter and anticipated data error in the aqueous humor VEGF and ranibizumab data (σ_1_ and σ_2_, respectively), we construct a weakly informed 10-dimensional prior probability distribution, P(θ), described using a hyperspace of independent truncated normal distributions (Supplementary Table S4.1). Ocular volumes have been excluded from the inference due to the consistent literature values for these parameters and the low model sensitivity to these parameters. Parameter sets were sampled using a differential evolution Markov chain Monte Carlo (DEMCMC) algorithm^63^ with 50 chains for 10,000 iterations. Initial values for *K*_*D*_ were sampled evenly across four orders of magnitude from the range *K*_*D*_ ∈ (10, 22,000). All inference was conducted using the probabilistic inference on noisy time-series framework^64^ (https://pints.readthedocs.io/en/latest/index.html). The open source code used to produce these results can be found at https://github.com/jcrawshaw1992/TwoCompartmentModel. For further descriptions of Bayesian inference, see Lambert.^65^

### Experimental Data

In our analysis, we consider the time-series pharmacokinetic data extracted from the aqueous humor of healthy cynomolgus macaques published by Niwa et al.^41^ (figs. 1 and 3 therein). The experimental procedure is detailed in Niwa et al.^41^; however, in the interest of completeness, we briefly summarize the relevant experimental protocol here. A single bolus of ranibizumab (0.5 mg in 50 µL) was injected into the vitreous cavity of the right eye of three cynomolgus macaques. VEGF and ranibizumab concentrations were measured using an ELISA from samples extracted from the aqueous humor at days 0, 1, 3, 7, 14, 21, 28, 35, 42, 49, and 56. We extracted data from figures 1 and 3 from Niwa et al.^41^ using WebPlotDigitizer (distributed under GNU AGPL v3; Supplementary Table S5.1). Cynomolgus macaques were chosen as the animal model due to their similarity to humans in terms of VEGF structure and ocular morphology.

The lower limit of quantification (LLQ) of the ELISA was 9.0 pg/mL and 0.156 ng/mL for VEGF and ranibizumab, respectively.^41^ Although the existing literature offers numerous techniques for handling data beyond the limits of quantification, the impact of these approaches on the inferred results are unclear. Notably, during the period when VEGF falls below the LLQ (days 1–21), the parameters *k*_off_, *V*_in_, $k_{\text{el}}^{v}$ and *CL* exhibit heightened sensitivity (Fig. 2E). Therefore, careful consideration is required when choosing an approach for handling data points below the LLQ to reduce the error introduced by the limits of quantification (uncertainty type U2).

### Impact of Implementing Data Correction Techniques on the Inferred Parameter Posterior Distributions

To investigate how the chosen data correction technique influences the inferred posterior distribution in the context of this model, we compared the posterior distributions obtained from a synthetic data set with inference following correction using each of the three widely used data correction techniques^66^^,^^67^:

M5: Replace each data point below the LLQ with LLQ/2;
M6: Replace the first data point below the LLQ with LLQ/2 and remove subsequent data points below the LLQ; and
M7: Replace each data point below the LLQ with 0.

A synthetic data set was generated using a reference parameter set, **θ_ref_** (as shown by black vertical lines in Fig. 3), and a predefined additive noise given by σ_*N*_ = 0.25. Ten data points were uniformly sampled across 9 weeks, processed using each data correction technique (individually) and analyzed using Bayesian inference to determine whether the reference parameters values (θ_ref_) could be recovered. This process was repeated 10 times to obtain average results and reduce stochastic artefacts introduced by the additive noise.

Figure 3 shows the normalized average posterior distributions inferred from the synthetic data sets without data correction (top row) and using each data correction technique (M5–M7; rows 2–4). The reference parameters are shown by the black lines in each subplot. The M7 data correction technique (row 4) resulted in inferred posterior distributions most similar to those obtained using the raw synthetic data and mean values most similar to the reference parameter values. The inferred posterior distributions obtained following the implementation of M6 (row 3) are similar to those obtained following the implementation of M7, whereas the M5 data correction technique (row 2) provided slightly inferior posterior distributions. The inferred *K*_*D*_ posterior distribution seems to be uninfluenced by the choice of data correction technique (column 7). Parameters $k_{\text{el}}^{v}$ (column 2) and *k*_off_ (column 6) were the most affected by the implementation of the M5 data correction technique. This observation is unsurprising, given the heightened sensitivity indices associated with $k_{\text{el}}^{v}$ and *k*_off_ during the time frame when data are likely falling below the LLQ. In Supplementary Material S6, we further explore the impact of each data correction technique for a series of synthetic data sets of varying data density and noise, showing that M6 is preferable for data sets with high data density and high noise (20 data points with σ_*N*_ = 0.5), and M7 is preferable for data sets with fewer data points (⩽20 data points) (Supplementary Fig. S6.1). In light of this favorable comparison for M7, we use this data correction technique to consider values below the LLQ in the Niwa et al.^41^ data set before analysis to minimize uncertainty from U2. We note that Niwa et al.^41^ also used the M7 technique to correct data below the LLQ.

### The PK/PD Parameter Estimates Inferred Using a Bayesian Approach Show a High Degree of Certainty, With the Exceptions of $k_{\text{el}}^{v}$ and *k*_off_

We analyzed the Niwa et al.^41^ data set using Bayesian inference with the model described in the Methods after preprocessing using data correction technique M7. Figure 4 shows the posterior distributions for each model parameter along the diagonal and pairwise marginal posterior distributions. A summary of the inference results is shown in Table 2. The convergence of the DEMCMC algorithm was confirmed using the rank-normalised $\hat{R}$ diagnostic test (Table 2). The marginal posteriors for $k_{\text{el}}^{r}$, $k_{\text{el}}^{h}$, *V*_in_, *K*_*D*_, and *CL* seem to follow Gaussian-like distributions, as shown in Figure 4. The $k_{\text{el}}^{r}$ marginal posterior exhibits low uncertainty, centred around 0.293 with a standard deviation (SD) of 0.007 (Table 2). This tight posterior distribution recovered for $k_{\text{el}}^{r}$ is consistent with the high model sensitivity. In contrast, the $k_{\text{el}}^{v}$ posterior distribution is wide with an interquartile range of [0.413 − 0.717] day^−1^, thus providing limited information about the VEGF elimination rate constant (Fig. 4, column 2). This possibly stems from the low sensitivity of $k_{\text{el}}^{v}$ to both VEGF and ranibizumab data (Fig. 2). A comparison of the inferred posterior distributions after the implementation of each data correction technique for the Niwa et al.^41^ data set is presented in Supplementary Figure S7.1. This analysis shows that the choice of data correction technique has little impact on most model parameters, with the notable exception of $k_{\text{el}}^{v}$ and *k*_off_, where we see that the data correction technique M6 produces a negative skew to the $k_{\text{el}}^{v}$ and *k*_off_ posterior distributions.

**Table 2.** Summary Statistics for the Marginal Posterior Distributions for Each Model Parameter Inferred Using Bayesian Inference Technique with the two-Compartment Model. We See the Mean ± SD for Each Parameter in Column 2. Column 3 Shows the Fisher-Pearson Coefficient of Skewness. Columns 4 and 5 Show the Interquartile Range (IQR; [25%, 50%, 75%]), and DEMCMC Chain Conversion (as Given by the $\hat{R}$ Value). The Right Most Column Shows the Units for Each Parameter

Parameter	Mean ± SD	Skew	IQR	$\hat{R}$	Units
$k_{\text{el}}^{r}$	0.293 ± 0.007	0.102	[0.288, 0.293, 0.297]	1.009	day^−1^
$k_{\text{el}}^{v}$	0.575 ± 0.188	0.163	[0.413, 0.548, 0.717]	1.005	day^−1^
$k_{\text{el}}^{c}$	0.259 ± 0.030	−1.171	[0.244, 0.266, 0.281]	1.009	day^−1^
$k_{\text{el}}^{h}$	0.176 ± 0.055	−0.131	[0.136, 0.179, 0.219]	1.007	day^−1^
*k*_off_	1.669 ± 1.174	1.069	[0.716, 1.448, 2.392]	1.009	day^−1^
*K*_*D*_	1459 ± 98	0.008	[1393, 1459, 1525]	1.007	pM
*CL*	2.505 ± 0.382	0.450	[2.474, 2.731, 3.351]	1.007	mL· day^−1^
*V*_in_	5.408 ± 0.856	0.502	[4.825, 5.332, 5.921]	1.007	pM·mL · day^−1^
σ_1_	0.198 ± 0.059	1.400	[0.187, 0.225, 0.344]	1.014	pM
σ_2_	0.143 ± 0.040	1.382	[0.136, 0.163, 0.243]	1.011	pM

The fitted time-series models for every species in each compartment are shown in Figure 5. The final 1000 parameter sets were drawn from the posterior distribution to determine a mean (blue) and SD (gray) for each molecular species at each time point. We see good agreement between the Niwa et al.^41^ data set (represented as black circles with black error bars) and the model fit. The large variability we observe in the vitreous VEGF equilibrium (Fig. 5A) is a consequence of the inverse relationship between the vitreous VEGF equilibrium and $k_{\text{el}}^{v}$ (Equation (S1.3)). The $k_{\text{el}}^{v}$ posterior distribution has a wide spread with a large degree of uncertainty (Fig. 4) resulting in a high degree of uncertainty in the simulated vitreous VEGF equilibrium, as evident by the large SD in Figure 5A. A more informative $k_{\text{el}}^{v}$ posterior distribution could be obtained if equilibrium vitreous VEGF data were available. Supplementary Figure F2 shows the posterior and prior distributions for each parameter alongside the DEMCMC traces.

### Inferred Intraocular Half-Lives for Each Molecular Species

Using the relationship between the half-life, *t*_1/2_, and the elimination rate constant, *k*_el_,

(1) $$\begin{array}{c} t_{1/2}=\frac{\ln(2)}{k_{\text{el}}},\; \end{array}$$

we obtain half-life posterior distributions for each species (Figs. 6A–D). The vertical black dashed line in Figure 6A indicates the ranibizumab half-life estimate calculated by Niwa et al.,^41^ while the gray distribution represents the mean ± SD ranibizumab half-life values reported in the literature, as summarised by Caruso et al.^68^ The Niwa et al.^41^ ranibizumab half-life calculation assumes that ranibizumab is eliminated exponentially and neglects the pharmacodynamic action of ranibizumab. By neglecting the pharmacodynamic action, the extended retention produced by the interaction between ranibizumab and VEGF is ignored, likely resulting in the underestimation of $t_{1/2}^{r}$, as is evident upon comparison with our inferred distributions (Fig. 6A). Using this $t_{1/2}^{r}$ value calculated by Niwa et al.^41^ and the Hutton-Smith et al.^39^ scaling relationship (Equation (S1.2)), we estimate $t_{1/2}^{v}$, $t_{1/2}^{c}$, and $t_{1/2}^{h}$, as shown by the dashed lines in Figures 6B–D. The estimates for $t_{1/2}^{c}$ and $t_{1/2}^{h}$ are in good agreement with the means of our inferred distributions (Figs. 6C–D); however, the estimate for $t_{1/2}^{v}$ differs notably from the mean of our inferred $t_{1/2}^{v}$ posterior distribution (Figs. 6B).

### Aqueous Humor VEGF Recovery Time Increases With Antibody Hydrodynamic Radius

Using the Stokes-Einstein relation, we can express the antibody hydrodynamic radius in terms of the molecular weight (MW)

(2) $$\begin{array}{c} R_{h}={\left(\frac{3\nu MW}{4\pi N_{A}}\right)}^{1/3},\; \end{array}$$

where *N*_*A*_ is Avogadro's number and ν is the partial specific volume of protein, taken as 0.73 cm^3^/g.^69^ As the elimination rate constant for ranibiuzmab is the most sensitive model parameter, and considering the relationships between the elimination rate constant, the molecular weight and the hydrodynamic radius given in Equations (S1.2) and (2), we investigated how the hydrodynamic radius of an anti-VEGF antibody affects the aqueous humor VEGF recovery time (PM2), assuming similar pharmacodynamic properties to ranibizumab. We calculated the elimination rate constants for this hypothetical antibody, as well as partially and fully bound VEGF, using Equation S1.2 and the mean inferred value of $k_{\text{el}}^{r}$ (Table 2). These rate constants, along with the optimized values for the other parameters (Table 2), were used to solve the two-compartment model to establish a theoretical relationship between PM2 and antibody hydrodynamic radius. Figure 6E illustrates that VEGF recovery time increases with increasing hydrodynamic radius in a near linear fashion.

### The Clearance Rate and VEGF Production Rate are Highly Correlated

The [*CL*, *V*_in_] marginal posterior distribution shown in Figure 4 is skewed and exhibits a pronounced aspect ratio, suggesting that a correlation exists between these parameters. A linear regression analysis on the final 1, 000 DEMCMC [*CL*, *V*_in_] samples demonstrates a strong relationship (*r*^2^ = 0.94) between *CL* and *V*_in_, given by

(3) $$\begin{array}{ccc} V_{\text{in}} & = & \lambda \cdot CL-\mu ,\; \end{array}$$

where λ = 2.34 pM and μ = 0.462 pM·mL · day^−1^.

Assuming that the initial aqueous humor VEGF sample (*V*_aq_[0] = 2.27 pM) accurately represents the steady-state concentration ($v_{\text{aq}}^{*}$), this relationship aligns well with the steady-state expression described in Equation (S1.3). Using the clearance rate constant values provided in the literature, the steady-state (Equation (S1.3)) may be used to estimate the VEGF production rate before treatment; however, this will require validation in human patients.

### On ELISA Measurements of Aqueous Humor VEGF

The ELISA technique used by Niwa et al.^41^ (Quantikine Human VEGF Immunoassay; R&D Systems, Minneapolis, MN, USA) is supposed to measure only free VEGF via locating the free binding sites on the VEGF molecules. However, both free and partially bound VEGF contain at least one free binding site, introducing the possibility of partially bound VEGF being captured in the free VEGF measurements. Moreover, competitive binding between the assay’s antibodies and ranibizumab may introduce confounding partially and fully bound VEGF in the free VEGF measurements.^42^ Sumner et al.^42^ reported that the binding affinity of the putative capture antibody of the Quantikine VEGF ELISA with VEGF is 33-fold stronger than the binding affinity between ranibizumab and VEGF, for a measured dissociation constant of *K*_*D*_ = 1.41 pM.^42^ Neglecting the potential contribution of partially and fully bound VEGF may introduce increased measurement uncertainty (type U3 uncertainty) and produce an overestimation of free VEGF. To address this, we now consider the free aqueous humor VEGF reported in the data as a linear combination of free, partially bound, and fully bound aqueous humor VEGF,

(4) $$\begin{array}{c} mv_{\text{aq}}\left(t\right)=v_{\text{aq}}\left(t\right)+\gamma c_{\text{aq}}\left(t\right)+\eta h_{\text{aq}}\left(t\right),\; \end{array}$$

where *mv*_aq_(*t*) is the measured total VEGF in the aqueous humor, and γ and η are the fractions of partially and fully bound aqueous humor VEGF measured by ELISA, respectively (γ ∈ [0, 1], η ∈ [0, 1]). To determine the fraction of partially and fully bound VEGF contained in the VEGF samples, we analyzed the Niwa et al.^41^ data set using Bayesian inference and the updated model assumption. The inferred posterior distributions for γ and η, along with each other model parameter, are shown in Figure 7A. The γ and η posteriors are heavily positively skewed with maxima at zero and long tails. Figures 7B and 7C show the optimized adapted model fits for the measured aqueous humor VEGF and ranibizumab, respectively. We note that the adapted model fit for measured VEGF is inferior to the original model fit shown in Figure 5E, with free VEGF overestimated between 1 and 6 weeks. This overestimation is caused by the presence of partially and fully bound VEGF, as seen in Figures 5E and 5F, respectively. The disparity between the experimentally measured VEGF and *mv*_aq_(*t*) during weeks 1 through 6 suggests it is unlikely that the measured free VEGF is corrupted by partially or fully bound VEGF in the Niwa et al.^41^ data set. From Table 3, we see that the disparity between the summary statistics inferred from this adapted model and the original model are within a half SD. We see that the inferred distribution for *K*_*D*_ is larger (1471 ± 299) using the adapted model in comparison to the original model (1459 ± 98), indicating that we have less certainty in the *K*_*D*_ posterior in the adapted model. We also see that the DEMCMC sampling exhibits inferior convergence in the adapted model (as given by the $\hat{R}$ values, Table 3). A detailed model comparison between the base and adapted model is presented in Supplementary Material S8.

**Table 3.** Summary Statistics for the Marginal Posterior Distributions for Each Model Parameter, Assuming Measured Aqueous Humor VEGF Is Given by *mv*_aq_(*t*) = *v*_aq_(*t*) + γ*c*_aq_(*t*) + η*h*_aq_(*t*). The Right Most Columns Show the Difference in the Mean and $\hat{R}$ Values for Each Parameter Compared to Those Inferred From the Full Model. γ and η Are Non-Dimensional Parameters Between [0, 1]

Parameter	Mean ± SD	IQR	$\hat{R}$	Units	ΔMean	$\Delta \hat{R}$
$k_{el}^{r}$	0.291 ± 0.007	[0.286, 0.291, 0.296]	1.031	day^−1^	−0.002	0.022
$k_{el}^{v}$	0.520 ± 0.184	[0.366, 0.471, 0.642]	1.027	day^−1^	−0.055	0.022
$k_{el}^{c}$	0.255 ± 0.031	[0.238, 0.262, 0.278]	1.030	day^−1^	−0.004	0.021
$k_{el}^{h}$	0.184 ± 0.052	[0.147, 0.187, 0.225]	1.032	day^−1^	0.008	0.025
*V*_in_	5.477 ± 0.888	[4.876, 5.397, 6.005]	1.027	pM·mL · day^−1^	0.069	0.020
*k*_off_	2.196 ± 1.412	[1.059, 1.982, 3.103]	1.025	day^−1^	0.527	0.016
*K*_*D*_	1471 ± 299	[1267, 1465, 1674]	1.027	pM	12	0.020
*CL*	2.560 ± 0.394	[2.296, 2.522, 2.792]	1.028	mL · day^−1^	0.055	0.021
γ	0.169 ± 0.151	[0.054, 0.126, 0.239]	1.036	nondim	—	—
η	0.045 ± 0.042	[0.015, 0.034, 0.064]	1.044	nondim	—	—
σ_1_	0.234 ± 0.074	[0.181, 0.219, 0.270]	1.040	pM	0.036	0.026
σ_2_	0.142 ± 0.037	[0.116, 0.135, 0.162]	1.033	pM	−0.001	0.022

The mean of the γ posterior distribution is higher and has a wider spread than that of η, suggesting that any contamination from non-free VEGF in the measurements is more likely due to partially bound VEGF. Consequently, we assessed the impact of the Quantikine VEGF ELISA capture antibody binding only to free and partially bound VEGF (η = 0). To quantify this, we derived the profile likelihood from a reduced model where γ was fixed within the range [0, 1], with η = 0. We repeated the inference process to optimize the remaining parameters for each fixed γ value. This allowed us to evaluate the decline in model fit, measured by the log-likelihood, as γ increases (Fig. 7D). The measured VEGF aqueous model fit for γ = 0 (red), 0.5 (blue), and 1 (green) are plotted in Figure 7E (with η = 0), showing that the best fit is achieved for γ = 0. As γ increases, the VEGF data points on days 14 and 21 are not adequately accounted for, compromising the model fit. If partially bound VEGF was present in the ELISA measurements, we would expect the VEGF data points on days 14 and 21 to be elevated, replicating the increase in partially bound VEGF (Fig. 5G). However, we do not see this, suggesting that there is little to no partially bound VEGF captured by the ELISA technique. Supplementary Figure S8.2 shows the posterior and prior distribution for each parameter alongside the DEMCMC traces. A more detailed comparison of the base model and the adapted model is shown in Supplementary Figures S8.3 and S8.5.

### The Transition Region of the VEGF Curve Is the Most Important Region to Sample

The parameter sensitivity at distinct segments of the aqueous humor VEGF curve (Fig. 2) and the discernible shift in the posterior distribution of *k*_off_ and $k_{\text{el}}^{v}$ following the exclusion of data points below the LLQ (M6; Fig. 3, row 3) suggest that the data collection schedule may play an important role in determining the uncertainty in the inferred posterior distributions from a given data set (type U4 uncertainty). Understanding which data regions have a notable impact on the uncertainty in the inferred parameters is important when designing experiments to study the PK/PD of novel anti-VEGF intraocular therapeutics. In this section, we explore the impact of U4 uncertainty by using synthetic data (σ_*N*_ = 2.5 × 10^−3^ with 10 data points) to consider how removing:

P1: The initial VEGF data point;
P2: The region of VEGF suppression;
P3: The VEGF transition region; and
P4: The VEGF return to equilibrium.

from the data impacts the posterior distributions of the model parameters. The four phases (P1–P4) of the aqueous humor VEGF curve are annotated in Figure 2A. We consider 10 synthetic data sets to ameliorate any random effects introduced using randomly generated synthetic data. Figure 8A shows the normalised average posterior distributions obtained from the synthetic data sets with each of the aforementioned regions removed. The black line in each subplot shows the input parameter value used to generate the synthetic data. Removal of the transition region (P3, Fig. 8A row 4) had the most notable impact on the posterior distributions. The exclusion of this region resulted in wider posteriors for $k_{\text{el}}^{v}$ and $k_{\text{el}}^{c}$ and notably skewed the $k_{\text{el}}^{h}$ posterior. This suggests that the transition region plays a critical role in constraining the estimation of these elimination rate constants. When P3 is removed, the model fit has a large SD between weeks 4 and 7, producing an apparent rebound in predicted aqueous humor VEGF before returning to a steady-state (Fig. 8A, final column, row 4).

Following the removal of the initial VEGF data point (P1, Fig. 8A row 2), we observed a slight skewing of *CL* and *V*_in_, consistent with our expectations based on the steady-state calculations in Equations S1.3. However, this effect was less pronounced than anticipated given the strong correlation between *CL* and *V*_in_ and the initial aqueous humor VEGF concentration. The removal of the final equilibrium region (P4, Fig. 8A row 5) had minimal impact on the posterior distributions, suggesting that this region has limited influence. It is likely that the data points from the transition region and the initial VEGF data point compensate for the absence of the final data points, contributing to the overall robustness of parameter estimation. Of note, the removal of both the initial and final points shown in Figure 8A row 5 (P1 and P4) did not yield notable effects on the posterior distributions, again indicating that the data points from the end of the transition region carry sufficient information to compensate for the lack of equilibrium data.

### Increased Information From the Transition Region

As the VEGF transition region (P3) is the most important region of the data set, as depicted in Figure 8A, we now explore the extent to which increasing data density in this region can provide additional information. To do this, we generated 10 sets of synthetic data with the number of data points in this region set at 0, 1, 3, or 5 and examined the average normalized posterior distributions, as shown in Figure 8B. With only a single data point in this region (*N*1, Fig. 8B row 2) we see a notable contraction in the posterior distributions for the elimination rate constants, as well as improved model fit, as compared with the synthetic data set without data points in the transition region (*N*0, Fig. 8B row 1). The posterior distributions for $k_{\text{el}}^{r}$, $k_{\text{el}}^{v}$, $k_{\text{el}}^{c}$, and $k_{\text{el}}^{h}$ and the model fits SD (rightmost column) continue to narrow as the number of data points in the transition region increases to 3 and then 5. This narrowing of posterior distributions provides reduced uncertainty and an increased confidence in the inferred mean as the available information in the transition region increases. The impact of increasing the number of data points in the transition region seems to be more important than the choice of data correction technique implemented (Figs. 3 and 8).

## Discussion

The semi-mechanistic two-compartment model of IVT ranibizumab treatment, originally presented by Hutton-Smith et al.,^39^ adequately and accurately describes the aqueous humor VEGF and ranibizumab profiles following a single IVT bolus of ranibizumab in the cynomolgus macaque (Fig. 5). Using this model, we implemented a Bayesian framework to analyze the data set extracted from Niwa et al.^41^ and to understand how the uncertainty in the data introduced by the lower limit of quantification (U2); the assumption that only free VEGF is measured by the ELISA technique (U3); and the data collection schedule (U4) contributed to uncertainty in the inferred parameter posteriors. The results from our analysis of the uncertainty suggest data correction technique M7 produced reduced uncertainty arising from the LLQ in the context of our model (Fig. 3) and that data collection schedules should prioritise data collection in the transition region of the aqueous humor VEGF concentration curve (Fig. 8). From our analysis, we showed that the posterior distribution of the dissociation constant (*K*_*D*_) has a mean and SD of 1459 ± 98 pM (Fig. 4; Table 2); the inferred aqueous humor clearance rate (*CL*) accurately reflects experimental values reported in the literature (Fig. 3; Table 2); and we recover the ranibizumab half-life ($t_{1/2}^{r}$) with satisfactory agreement with previously reported values in the literature (Fig. 6). Moreover, we were able to recover an estimate of the retinal VEGF production rate (*V*_in_), which is otherwise unobtainable through experimental approaches (Equation (3)).

Previous model fitting studies using in vivo data sets have reported a dissociation constant of *K*_*D*_ = 21, 326 pM^39^ and *K*_*D*_ = 19,000 pM,^40^ each an order of magnitude larger than the mean *K*_*D*_ obtained in this study. These studies, using human data, were confined to aqueous humor VEGF samples and concluded that deriving accurate *K*_*D*_ estimates was challenging in the absence of aqueous humor ranibizumab time-series data.^39^^,^^40^ To address the absence of aqueous humor ranibizumab data, Hutton-Smith et al.^39^^,^^40^ iteratively implemented a least-squares optimization process using a series of fixed *K*_*D*_ values to estimate $t_{\text{1/2}}^{r}$ and *V*_in_ for each value of *K*_*D*_. The estimated $t_{\text{1/2}}^{r}$ that most closely aligned with experimental values served as the basis to predict *K*_*D*_. Our Bayesian approach allows for direct inference of *K*_*D*_ and allowed us to express the uncertainty in the data in terms of uncertainty in the parameter posterior distributions. As VEGF is conserved between humans and cynomolgus macaques, the observed disparity in *K*_*D*_ between studies^39^^,^^40^ likely arises from differences in the data analysis techniques used and the absence of ranibizumab data in the human data sets. Experimental estimates for *K*_*D*_ vary considerably across the literature (Table 1) and depend on the experimental techniques used.^24^^–^^40^ The weakly informative *K*_*D*_ prior distribution was constructed to reflect the uncertainty in the experimental literature (Supplementary Table S4.1). Despite this, we obtained a relatively narrow inferred posterior distribution for *K*_*D*_, suggesting a high degree of confidence for the parameter inference associated with the Niwa et al.^41^ data set. The conservation of VEGF between humans and cynomolgus macaques suggests that the mean of our inferred *K*_*D*_ distribution (Fig. 4) may be a useful *K*_*D*_ value in human studies; however, validation with aqueous humor ranibizumab data collected from clinical studies will be necessary to confirm this.

A global sensitivity analysis revealed that aqueous humor ranibizumab half-life and VEGF recovery time are most sensitive to $k_{\text{el}}^{r}$ (Fig. 2). Considering the relationships between $k_{\text{el}}^{i}$, molecular weight and hydrodynamic radius for a molecular species *i* given in Equations (2) and (S1.2), our sensitivity analysis is in agreement with previous experimental studies that identify the hydrodynamic radius of macromolecules as the primary determinant of their ocular retention.^70^^,^^68^ We further tested this by establishing a numerical relationship between VEGF recovery time and hydrodynamic radius (Fig. 6E), showing that VEGF recovery time is extended with increasing hydrodynamic radius in an approximately linear manner. This result is consistent with results from Caruso et al.,^68^ who used experimental data to show that the half-life increases linearly with hydrodynamic radius in several species, including human and cynomolgus macaque. This theoretical relationship between VEGF recovery time and antibody hydrodynamic radius (Fig. 6E) is highly relevant to the development of novel therapies. However, it is also suspected that the efficiency of antibody diffusion through the retina to the choroid decreases with increasing hydrodynamic radius.^71^ The results presented in this study assume that retinal drug absorption is not the primary clearance route. This assumption may alter the relative importance seen in $k_{\text{el}}^{r}$ and hydrodynamic radius and hence considering the role of retinal absorption will be important to gain a better understanding of the relationship between hydrodynamic radius and VEGF recovery time and is left for future work.

The strong agreement between the inferred posterior distributions for $k_{\text{el}}^{r}$, $k_{\text{el}}^{c}$, and $k_{\text{el}}^{h}$ with the values estimated using the Stokes-Einstein relation (Fig. S1.2) provides confidence in results built on the assumption of spherical molecular geometries. The wide posterior distribution for $k_{\text{el}}^{v}$ in Figure 4 suggests a lack of practical identifiability for this parameter in the current data set or the presence of an overlooked underlying process. Indeed, experimental evidence suggests that there may be additional unidentified processes influencing VEGF dispersion in the vitreous.^72^ Using Equation (1), we obtained posterior distributions for the half-life of each species. The calculated $t_{1/2}^{r}$ by Niwa et al.^41^ slightly underestimated our inferred mean $t_{1/2}^{r}$, likely owing to their omission of binding kinetics in their calculations, potentially leading to an underestimation of retention time. To the best of our knowledge, we are the first to directly infer the elimination rate constants for each molecular species for IVT ranibizumab treatment.

A regression analysis using samples taken from the marginal posterior distribution of *V*_in_ and *CL*, as shown in Figure 4, successfully reproduced the relationship described in Equation (S1.3) and recovered the initial aqueous humor VEGF concentration with excellent agreement with the data. The straightforward relationship described by Equation (3) could offer a patient-specific prediction of the retinal VEGF production rate based on a single aqueous humor VEGF sample from a naive eye. However, it is important to note that this relationship was established using data from healthy macaques. The introduction of additional factors during retinal disease in human patients, such as potentially time-varying VEGF production rates and treatment scheduling, may challenge the applicability of this relationship. Therefore, future work is necessary to assess the validity of this prediction in the context of patient data.

Recently, Sumner et al.^42^ presented evidence to suggest that the ELISA technique (Quantikine Human VEGF Immunoassay; R&D Systems, Minneapolis, MN, USA) measures bound VEGF as part of the VEGF measurement in samples containing both VEGF and anti-VEGF antibodies, such as ranibizumab. This raises the concern that the free VEGF measurements from Niwa et al.,^41^ who used the same commercially available R&D Systems Quantikine Human VEGF ELISA kit, may incorporate some unknown fraction of partially and fully bound VEGF, introducing a potential source of uncertainty, of type U3. To investigate this, we adapted the base model with the assumption that the VEGF measurement contains a proportion, γ, of partially bound VEGF and a proportion, η, of fully bound VEGF. Our analysis shows that the proportionality constants γ and η are skewed with peaks at zero (Fig. 7A). Moreover, our results show that the adapted model provides an inferior fit compared to the original model (Fig. 7D and Supplementary Fig. S8.5), suggesting that the contribution of partially and fully bound VEGF in the Quantikine VEGF ELISA are minimal in this data set. These results are contrary to those reported by Sumner et al.^42^ One possibility for this difference may be differences in the incubation period between the studies, with Sumner et al.^42^ showing that the incubation period is an important factor when considering the impact of competitive binding. Further work is necessary to fully quantify the impact of competitive binding between the putative capture antibody of the Quantikine VEGF ELISA and ranibizumab in aqueous humor samples.

We investigated the impact of the lower limit of quantification (LLQ) and the data collection schedule on data uncertainty. We found that data collected in the transition region of the VEGF curve (P3, Fig. 2A) had the most significant effect on parameter estimation. Excluding P3 data increased uncertainty in the posterior distributions, whereas denser P3 sampling progressively reduced uncertainty, particularly for elimination rate constants. This result is anticipated because P3 is a dynamic phase of the concentration profile. Excluding data points in the suppressed region of the VEGF curve (P2) had minimal impact on the inferred posterior distributions. As P2 data fell below the LLQ in this study, we assessed several data correction techniques using synthetic data sets. For data sets with fewer than 20 points (such as the Niwa et al.^41^ data set), the M7 data correction technique, as used by Niwa et al.,^41^ had the smallest impact, whereas for noisy data with higher sampling densities (>20 data points with the synthetic data noise SD given by σ_*N*_ = 0.5), removing data below the LLQ (M6) was optimal (Supplementary Fig. S6.1). This outcome again suggests that having sufficient data in the transition region compensates for the absence of data in the suppressed region and excluding LLQ-affected data reduces error in parameter estimations. Across all data densities, M5 performed the least effectively, likely overestimating aqueous humor VEGF in P2. These findings imply that prioritising detailed data collection in the transition region (P3) reduces the uncertainly in the inferred posterior distributions. The approximate timing of the transition region for novel anti-VEGF therapeutics can be predicted using antibody specific PK/PD models and the scaling relationship between elimination rate constants provided by Hutton-Smith et al.^39^ (Equation (S1.2)). As such, this analysis provides theoretical guidelines to enhance PK/PD parameter estimation through improved data collection schedules in both human and animal subjects.

In summary, the semi-mechanistic two-compartment model of IVT ranibizumab treatment considered in this study accurately describes the profiles of aqueous humor VEGF and ranibizumab after a single IVT bolus in cynomolgus macaques. Our inferred parameter posterior distributions align well with the values in the literature when available, with the exception of *K*_*D*_, for which we inferred a comparatively narrow distribution with a mean of 1459 pM and a SD of 98 pM (Fig. 4; Table 2). We found that this model was most sensitive to the ranibizumab elimination rate (Fig. 2), which is known to be related to the molecular weight of ranibizumab (Equation (S1.2)). Further examination showed a near linear increase in VEGF suppression time with increased antibody hydrodynamic radius (Fig. 6E). Notably, data correction technique M7 and data collection in the transition region of the VEGF curve emerged as a crucial strategy to reduce uncertainty in the posterior distributions (Figs. 3 and 8). Lastly, our findings indicate that partially bound VEGF likely has a negligible contribution to the ELISA measurement (Fig. 7 and Table 3).

## Supplementary Material

Supplement 1
