# Supplementary material for: Modeling the Ocular Pharmacokinetics and Pharmacodynamics of Ranibizumab for Improved Understanding and Data Collection Strategies in Ocular Diseases
Source: Invest Ophthalmol Vis Sci. 2025 Jun 6;66(6):20. doi: 10.1167/iovs.66.6.20 (PMC12155695; doi:10.1167/iovs.66.6.20)
Supplement: Supplement 1 [file iovs-66-6-20_s001.pdf]

Supplementary Material

S1. A semi-mechanistic model of ranibizumab ocular PK/PD

Here we provide further details on the semi-mechanistic two-compartment model of ocular VEGF suppression after a single IVT ranibizumab treatment, originally presented by Hutton-Smith *et al.*[1] and described in the Methods section of the main manuscript. Owing to the short diffusion time of large molecules in comparison to the half-life of each species, we assume that the system is well-mixed.[1] Consequently, this system can be described using a system of coupled non-linear ordinary differential equations

$$
\begin{aligned}
&\textbf{Vitreous}\\
&\frac{dv_{\text{vit}}}{dt} = (k_{\text{off}}c_{\text{vit}} - 2k_{\text{on}}v_{\text{vit}}r_{\text{vit}}) - k^{v}_{\text{el}}v_{\text{vit}} + \frac{V_{\text{in}}}{\mathcal{V}_{\text{vit}}},\\
&\frac{dr_{\text{vit}}}{dt} = (k_{\text{off}}c_{\text{vit}} - 2k_{\text{on}}v_{\text{vit}}r_{\text{vit}}) + (2k_{\text{off}}h_{\text{vit}} - k_{\text{on}}r_{\text{vit}}c_{\text{vit}}) - k^{r}_{\text{el}}r_{\text{vit}},\\
&\frac{dc_{\text{vit}}}{dt} = -(k_{\text{off}}c_{\text{vit}} - 2k_{\text{on}}v_{\text{vit}}r_{\text{vit}}) + (2k_{\text{off}}h_{\text{vit}} - k_{\text{on}}r_{\text{vit}}c_{\text{vit}}) - k^{c}_{\text{el}}c_{\text{vit}},\\
&\frac{dh_{\text{vit}}}{dt} = -(2k_{\text{off}}h_{\text{vit}} - k_{\text{on}}r_{\text{vit}}c_{\text{vit}}) - k^{h}_{\text{el}}h_{\text{vit}},\\
&\textbf{Aqueous}\\
&\frac{dv_{\text{aq}}}{dt} = (k_{\text{off}}c_{\text{aq}} - 2k_{\text{on}}v_{\text{aq}}r_{\text{aq}}) + \frac{\mathcal{V}_{\text{vit}}}{\mathcal{V}_{\text{aq}}}k^{v}_{\text{el}}v_{\text{vit}} - \frac{CL}{\mathcal{V}_{\text{aq}}}v_{\text{aq}},\\
&\frac{dr_{\text{aq}}}{dt} = (k_{\text{off}}c_{\text{aq}} - 2k_{\text{on}}v_{\text{aq}}r_{\text{aq}}) + (2k_{\text{off}}h_{\text{aq}} - k_{\text{on}}r_{\text{aq}}c_{\text{aq}}) + \frac{\mathcal{V}_{\text{vit}}}{\mathcal{V}_{\text{aq}}}k^{r}_{\text{el}}r_{\text{vit}} - \frac{CL}{\mathcal{V}_{\text{aq}}}r_{\text{aq}},\\
&\frac{dc_{\text{aq}}}{dt} = -(k_{\text{off}}c_{\text{aq}} - 2k_{\text{on}}v_{\text{aq}}r_{\text{aq}}) + (2k_{\text{off}}h_{\text{aq}} - k_{\text{on}}r_{\text{aq}}c_{\text{aq}}) + \frac{\mathcal{V}_{\text{vit}}}{\mathcal{V}_{\text{aq}}}k^{c}_{\text{el}}c_{\text{vit}} - \frac{CL}{\mathcal{V}_{\text{aq}}}c_{\text{aq}},\\
&\frac{dh_{\text{aq}}}{dt} = -(2k_{\text{off}}h_{\text{aq}} - k_{\text{on}}r_{\text{aq}}c_{\text{aq}}) + \frac{\mathcal{V}_{\text{vit}}}{\mathcal{V}_{\text{aq}}}k^{h}_{\text{el}}h_{\text{vit}} - \frac{CL}{\mathcal{V}_{\text{aq}}}h_{\text{aq}},
\end{aligned}
\tag{S1.1}
$$

where $v_{\text{vit,aq}}$, $r_{\text{vit,aq}}$, $c_{\text{vit,aq}}$, $h_{\text{vit,aq}}$ describe the concentration of free VEGF, unbound ranibizumab, the VEGF-ranibizumab complex (VR) and ranibizumab-VEGF-ranibizumab complex (RVR), respectively, in the vitreous (vit) and the aqueous humor (aq) as summarised in Table S1.1. The vitreous and aqueous humor volumes are given by $\mathcal{V}_{\text{vit}}$ and $\mathcal{V}_{\text{aq}}$, respectively. The elimination rate constants for V, R, VR and RVR are given by k^{v}_{el}, k^{r}_{el}, k^{c}_{el}, k^{h}_{el}, respectively. Using the Stokes-Einstein relationship, Hutton-Smith *et al.*[1] established a scaling relationship between the elimination rate constants of molecular species i and j

$$k^{i}_{\text{el}} = \left(\frac{MW_j}{MW_i}\right)^{1/3} k^{j}_{\text{el}}, \tag{S1.2}$$

where MW is the molecular weight. The Stokes-Einstein relationship assumes, among other things, that the molecules can be approximated as spheres, and shows that the diffusion decreases as molecular weight increases.

Initial conditions

The initial concentration of ranibizumab in the vitreous and aqueous humor are given by

$$r_{\text{vit}}(0) = \frac{D_0}{\mathcal{V}_{\text{vit}}}\frac{1}{MW_R} \text{ and } r_{\text{aq}}(0) = 0,$$

Table S1.1: Molecular species, notations and molecular weights. All concentrations are measured in pM and are a function of time. Concentrations are shown for the vitreous (vit) and aqueous humor (aq), as denoted by the subscript. Table adapted from Hutton-Smith *et al.*[1]

Molecular species	Notation	Molecular weight (kDa)	Concentration (pM)
VEGF	V	44.00[2]	$v_{\text{vit}},\ v_{\text{aq}}$
Ranibizumab	R	48.35[3]	$r_{\text{vit}},\ r_{\text{aq}}$
VEGF-ranibizumab complex	VR	88.35	$c_{\text{vit}},\ c_{\text{aq}}$
Ranibizumab-VEGF-ranibizumab complex	RVR	136.70	$h_{\text{vit}},\ h_{\text{aq}}$

respectively, where $D_0 = 0.5$ mg is the dose of ranibizumab delivered at time $t = 0$, and MW_R is the molecular weight of ranibizumab. We assume that the spatial homogenisation of ranibizumab occurs on a time scale much faster than that captured by the model and that the aqueous humor has not previously been exposed to ranibizumab. We also assume that the initial concentrations of VEGF in the vitreous and aqueous humor are given by the steady-state concentrations in each compartment (v^*_{vit} and v^*_{aq}, respectively)

$$v_{\text{vit}}(0) = v^*_{\text{vit}}, \text{ and } v_{\text{aq}}(0) = v^*_{\text{aq}}.$$

Finally, the initial conditions for each complex in both compartments are given by

$$c_{\text{vit}}(0) = 0,\ h_{\text{vit}}(0) = 0,\ c_{\text{aq}}(0) = 0, \text{ and } h_{\text{aq}}(0) = 0.$$

Following the complete elimination of ranibizumab, the system is assumed to return to a steady-state ($r_{\text{vit,aq}} = c_{\text{vit,aq}} = h_{\text{vit,aq}} = 0$). By considering the steady-state of the system, we obtain the following expressions for the steady-state vitreous and aqueous humor VEGF concentrations:

$$v^*_{\text{vit}} = \frac{V_{\text{in}}}{k^v_{\text{el}} \mathcal{V}_{\text{vit}}} \text{ and } v^*_{\text{aq}} = \frac{V_{\text{in}}}{CL}. \tag{S1.3}$$

The structural identifiability of this model has been confirmed, to within the limits of this method, using the simple scaling method presented by Castro and de Boer,[4] and can be found in Supplementary Material S2.

S2. Confirming Structural Identifiability

We followed the method presented by Castro and de Boer,[4] utilising invariances of the ODE system described in Supplementary Material S1 under the parameter scaling transformations. We begin by rewriting the coupled equations as a linear system

$$\frac{dx_i}{dt} = f_i(\mathbf{X}; \boldsymbol{\lambda}),$$

where $\mathbf{X}$ and $\boldsymbol{\lambda}$ describe the variable set and the parameter set, respectively, as follows;

$$\mathbf{X} = \{v_{\text{aq}}, r_{\text{aq}}, v_{\text{vit}}, r_{\text{vit}}, c_{\text{vit}}, h_{\text{vit}}, c_{\text{aq}}, h_{\text{aq}}\} \quad \text{and} \quad \boldsymbol{\lambda} = \{k_{\text{off}}, k_{\text{on}}, V_{\text{in}}, \mathcal{V}_{\text{vit}}, k^v_{\text{el}}, k^r_{\text{el}}, k^c_{\text{el}},\ k^h_{\text{el}}, \mathcal{V}_{\text{aq}}, CL\}.$$

We can describe f_i as a sum of M linearly independent components,

$$\frac{dx_i}{dt} = \sum_{k=1}^{M} f_{i,k}(\tilde{\boldsymbol{X}}_k, \tilde{\boldsymbol{\lambda}}_k). \tag{S2.1}$$

where $\tilde{\boldsymbol{X}}_k$ and $\tilde{\boldsymbol{\lambda}}_k$, describe respectively the subset of variables and parameters upon which the function $f_{i,k}$ is dependent. To test the model invariance under scaling, we now scale each unmeasured parameter and unmeasured variable using $\boldsymbol{u}_{\tilde{\boldsymbol{X}}_k}$ and $\boldsymbol{u}_{\tilde{\boldsymbol{\lambda}}_k}$, respectively,

$$\begin{aligned} \frac{dx_i}{dt} &= \sum_{k=1}^{M} f_{i,k}(\boldsymbol{u}_{\tilde{\boldsymbol{X}}_k}\tilde{\boldsymbol{X}}_k, \boldsymbol{u}_{\tilde{\boldsymbol{\lambda}}_k}\tilde{\boldsymbol{\lambda}}_k), \qquad i = 4, \text{ and } 5, \\ \frac{dx_i}{dt} &= \frac{1}{u_{x_i}}\sum_{k=1}^{M} f_{i,k}(\boldsymbol{u}_{\tilde{\boldsymbol{X}}_k}\tilde{\boldsymbol{X}}_k, \boldsymbol{u}_{\tilde{\boldsymbol{\lambda}}_k}\tilde{\boldsymbol{\lambda}}_k), \quad i = 1,2,3,6,7, \text{ and } 8. \end{aligned} \tag{S2.2}$$

The trivial solution is $\boldsymbol{u}_{\tilde{\boldsymbol{X}}_k} = \boldsymbol{u}_{\tilde{\boldsymbol{\lambda}}_k} = \boldsymbol{1}$. If the solution is invariant under this transformation, then the right hand side of Equation (S2.1) will be equal to the right hand side of Equation (S2.2). Using the functional linear independence of the functions $f_{i,k}$ we can split each summand

$$\begin{aligned} f_{i,k}(\tilde{\boldsymbol{X}}_k, \tilde{\boldsymbol{\lambda}}_k) &= f_{i,k}(\boldsymbol{u}_{\tilde{\boldsymbol{X}}_k}\tilde{\boldsymbol{X}}_k, \boldsymbol{u}_{\tilde{\boldsymbol{\lambda}}_k}\tilde{\boldsymbol{\lambda}}_k) \quad \text{for} \quad i = 4, \text{ and } 5, \\ f_{i,k}(\tilde{\boldsymbol{X}}_k, \tilde{\boldsymbol{\lambda}}_k) &= \frac{1}{u_{x_i}} f_{i,k}(\boldsymbol{u}_{\tilde{\boldsymbol{X}}_k}\tilde{\boldsymbol{X}}_k, \boldsymbol{u}_{\tilde{\boldsymbol{\lambda}}_k}\tilde{\boldsymbol{\lambda}}_k) \quad \text{for} \quad i = 1,2,3,6,7, \text{ and } 8. \end{aligned}$$

Each of the linearly independent functions in our system can be written as,

Vitreous compartment

$$\frac{dv_{\text{vit}}}{dt}: \; f_{1,1} = k_{\text{off}}c_{\text{vit}}, \quad f_{1,2} = 2k_{\text{on}}v_{\text{vit}}r_{\text{vit}}, \quad f_{1,3} = k_{\text{el}}^{v}v_{\text{vit}}, \quad f_{1,4} = \frac{V_{\text{in}}}{\mathcal{V}_{\text{vit}}},$$

$$\frac{dr_{\text{vit}}}{dt}: \; f_{2,1} = k_{\text{off}}c_{\text{vit}}, \quad f_{2,2} = 2k_{\text{on}}v_{\text{vit}}r_{\text{vit}}, \quad f_{2,3} = 2k_{\text{off}}h_{\text{vit}}, \quad f_{2,4} = k_{\text{on}}r_{\text{vit}}c_{\text{vit}}, \quad f_{2,5} = k_{\text{el}}^{r}r_{\text{vit}},$$

$$\frac{dc_{\text{vit}}}{dt}: \; f_{3,1} = k_{\text{off}}c_{\text{vit}}, \quad f_{3,2} = 2k_{\text{on}}v_{\text{vit}}r_{\text{vit}}, \quad f_{3,3} = 2k_{\text{off}}h_{\text{vit}}, \quad f_{3,4} = k_{\text{on}}r_{\text{vit}}c_{\text{vit}}, \quad f_{3,5} = k_{\text{el}}^{c}c_{\text{vit}},$$

$$\frac{dh_{\text{vit}}}{dt}: \; f_{4,1} = -2k_{\text{off}}h_{\text{vit}}, \quad f_{4,2} = k_{\text{on}}r_{\text{vit}}c_{\text{vit}}, \quad f_{4,3} = k_{\text{el}}^{h}h_{\text{vit}},$$

Aqueous compartment

$$\frac{dv_{\text{aq}}}{dt}: f_{5,1} = k_{\text{off}}c_{\text{aq}}, \quad f_{5,2} = 2k_{\text{on}}v_{\text{aq}}r_{\text{aq}}, \quad f_{5,3} = \frac{\mathcal{V}_{\text{vit}}}{\mathcal{V}_{\text{aq}}}k_{\text{el}}^{v}v_{\text{vit}}, \quad f_{5,4} = \frac{CL}{\mathcal{V}_{\text{aq}}}v_{\text{aq}},$$

$$\frac{dr_{\text{aq}}}{dt}: f_{6,1} = k_{\text{off}}c_{\text{aq}}, \quad f_{6,2} = 2k_{\text{on}}v_{\text{aq}}r_{\text{aq}}, \quad f_{6,3} = 2k_{\text{off}}h_{\text{aq}}, \quad f_{6,4} = k_{\text{on}}r_{\text{aq}}c_{\text{aq}}, \quad f_{6,5} = \frac{\mathcal{V}_{\text{vit}}}{\mathcal{V}_{\text{aq}}}k_{\text{el}}^{r}r_{\text{vit}}, \quad f_{6,6} = \frac{CL}{\mathcal{V}_{\text{aq}}}r_{\text{aq}},$$

$$\frac{dc_{\text{aq}}}{dt}: f_{7,1} = k_{\text{off}}c_{\text{aq}}, \quad f_{7,2} = 2k_{\text{on}}v_{\text{aq}}r_{\text{aq}}, \quad f_{7,3} = 2k_{\text{off}}h_{\text{aq}}, \quad f_{7,4} = k_{\text{on}}r_{\text{aq}}c_{\text{aq}}, \quad f_{7,5} = \frac{\mathcal{V}_{\text{vit}}}{\mathcal{V}_{\text{aq}}}k_{\text{el}}^{c}c_{\text{vit}}, \quad f_{7,6} = \frac{CL}{\mathcal{V}_{\text{aq}}}c_{\text{aq}},$$

$$\frac{dh_{\text{aq}}}{dt}: f_{8,1} = 2k_{\text{off}}h_{\text{aq}}, \quad f_{8,2} = k_{\text{on}}r_{\text{aq}}c_{\text{aq}}, \quad f_{8,3} = \frac{\mathcal{V}_{\text{vit}}}{\mathcal{V}_{\text{aq}}}k_{\text{el}}^{h}h_{\text{vit}}, \quad f_{8,4} = \frac{CL}{\mathcal{V}_{\text{aq}}}h_{\text{aq}}.$$

We now scale the linearly independent functions, denoted as $f^s_{i,k}$.

Scaled vitreous compartment

$$\frac{dv_{\text{vit}}}{dt}: \quad f^s_{1,1} = \frac{1}{u_{v_{\text{vit}}}} u_{k_{\text{off}}} k_{\text{off}} u_{c_{\text{vit}}} c_{\text{vit}}, \quad f^s_{1,2} = \frac{1}{u_{v_{\text{vit}}}} u_{k_{\text{on}}} k_{\text{on}} u_{v_{\text{vit}}} v_{\text{vit}} u_{r_{\text{vit}}} r_{\text{vit}}, \quad f^s_{1,3} = \frac{1}{u_{v_{\text{vit}}}} u_{k^v_{\text{el}}} k^v_{\text{el}} u_{v_{\text{vit}}} v_{\text{vit}},$$
$$f^s_{1,4} = \frac{1}{u_{v_{\text{vit}}}} \frac{u_{V_{\text{in}}} V_{\text{in}}}{u_{\mathcal{V}_{\text{vit}}} \mathcal{V}_{\text{vit}}}$$

$$\frac{dr_{\text{vit}}}{dt}: f^s_{2,1} = \frac{1}{u_{r_{\text{vit}}}} u_{k_{\text{off}}} k_{\text{off}} u_{c_{\text{vit}}} c_{\text{vit}}, \quad f^s_{2,3} = \frac{1}{u_{r_{\text{vit}}}} u_{k_{\text{off}}} k_{\text{off}} u_{h_{\text{vit}}} h_{\text{vit}}, \quad f^s_{2,4} = \frac{1}{u_{r_{\text{vit}}}} u_{k_{\text{on}}} k_{\text{on}} u_{r_{\text{vit}}} r_{\text{vit}} u_{c_{\text{vit}}} c_{\text{vit}},$$
$$f^s_{2,5} = \frac{1}{u_{r_{\text{vit}}}} u_{k^r_{\text{el}}} k^r_{\text{el}} u_{r_{\text{vit}}} r_{\text{vit}}$$

$$\frac{dc_{\text{vit}}}{dt}: f^s_{3,1} = \frac{1}{u_{c_{\text{vit}}}} u_{k_{\text{off}}} k_{\text{off}} u_{c_{\text{vit}}} c_{\text{vit}}, \quad f^s_{3,2} = \frac{1}{u_{c_{\text{vit}}}} u_{k_{\text{on}}} k_{\text{on}} u_{V_{\text{vit}}} V_{\text{vit}} u_{r_{\text{vit}}} r_{\text{vit}}, \quad f^s_{3,3} = \frac{1}{u_{c_{\text{vit}}}} u_{k_{\text{off}}} k_{\text{off}} u_{h_{\text{vit}}} h_{\text{vit}},$$
$$f^s_{3,4} = \frac{1}{u_{c_{\text{vit}}}} u_{k_{\text{on}}} k_{\text{on}} u_{r_{\text{vit}}} r_{\text{vit}} u_{c_{\text{vit}}} c_{\text{vit}}, \quad f^s_{3,5} = \frac{1}{u_{c_{\text{vit}}}} u_{k^c_{\text{el}}} k^c_{\text{el}} u_{c_{\text{vit}}} c_{\text{vit}}$$

$$\frac{dh_{\text{vit}}}{dt}: f^s_{4,1} = \frac{1}{u_{h_{\text{vit}}}} u_{k_{\text{off}}} k_{\text{off}} u_{h_{\text{vit}}} h_{\text{vit}}, \quad f^s_{4,2} = \frac{1}{u_{h_{\text{vit}}}} u_{k_{\text{on}}} k_{\text{on}} u_{r_{\text{vit}}} r_{\text{vit}} u_{c_{\text{vit}}} c_{\text{vit}}, \quad f^s_{4,3} = \frac{1}{u_{h_{\text{vit}}}} u_{k^h_{\text{el}}} k^h_{\text{el}} u_{h_{\text{vit}}} h_{\text{vit}}$$

Scaled aqueous compartment

$$\frac{dv_{\text{aq}}}{dt}: f^s_{5,1} = u_{k_{\text{off}}} k_{\text{off}} u_{c_{\text{aq}}} c_{\text{aq}}, \quad f^s_{5,2} = 2u_{k_{\text{on}}} k_{\text{on}} v_{\text{aq}} r_{\text{aq}}, \quad f^s_{5,3} = \frac{u_{\mathcal{V}_{\text{vit}}} \mathcal{V}_{\text{vit}}}{u_{\mathcal{V}_{\text{aq}}} \mathcal{V}_{\text{aq}}} u_{k^v_{\text{el}}} k^v_{\text{el}} u_{v_{\text{vit}}} v_{\text{vit}}, \quad f^s_{5,4} = \frac{u_{CL} CL}{u_{\mathcal{V}_{\text{aq}}} \mathcal{V}_{\text{aq}}} v_{\text{aq}}$$

$$\frac{dr_{\text{aq}}}{dt}: f^s_{6,1} = u_{k_{\text{off}}} k_{\text{off}} u_{c_{\text{aq}}} c_{\text{aq}}, \quad f^s_{6,2} = u_{k_{\text{on}}} k_{\text{on}} v_{\text{aq}} r_{\text{aq}}, \quad f^s_{6,3} = u_{k_{\text{off}}} k_{\text{off}} u_{h_{\text{aq}}} h_{\text{aq}}, \quad f^s_{6,4} = u_{k_{\text{on}}} k_{\text{on}} r_{\text{aq}} u_{c_{\text{aq}}} c_{\text{aq}},$$
$$f^s_{6,5} = \frac{u_{\mathcal{V}_{\text{vit}}} \mathcal{V}_{\text{vit}}}{u_{\mathcal{V}_{\text{aq}}} \mathcal{V}_{\text{aq}}} u_{k^r_{\text{el}}} k^r_{\text{el}} u_{r_{\text{vit}}} r_{\text{vit}}, \quad f^s_{6,6} = \frac{u_{CL} CL}{u_{\mathcal{V}_{\text{aq}}} \mathcal{V}_{\text{aq}}} r_{\text{aq}}$$

$$\frac{dc_{\text{aq}}}{dt}: f^s_{7,1} = \frac{1}{u_{c_{\text{aq}}}} u_{k_{\text{off}}} k_{\text{off}} u_{c_{\text{aq}}} c_{\text{aq}}, \quad f^s_{7,2} = \frac{1}{u_{c_{\text{aq}}}} u_{k_{\text{on}}} k_{\text{on}} v_{\text{aq}} r_{\text{aq}}, \quad f^s_{7,3} = \frac{1}{u_{c_{\text{aq}}}} u_{k_{\text{off}}} k_{\text{off}} u_{h_{\text{aq}}} h_{\text{aq}},$$
$$f^s_{7,4} = \frac{1}{u_{c_{\text{aq}}}} u_{k_{\text{on}}} k_{\text{on}} r_{\text{aq}} u_{c_{\text{aq}}} c_{\text{aq}} \quad f^s_{7,5} = \frac{1}{u_{c_{\text{aq}}}} \frac{u_{V_{\text{vit}}} V_{\text{vit}}}{u_{V_{\text{aq}}} V_{\text{aq}}} u_{k^c_{\text{el}}} k^c_{\text{el}} u_{c_{\text{vit}}} c_{\text{vit}}, \quad f^s_{7,6} = \frac{1}{u_{c_{\text{aq}}}} \frac{u_{CL} CL}{u_{V_{\text{aq}}} V_{\text{aq}}} u_{c_{\text{aq}}} c_{\text{aq}}$$

$$\frac{dh_{\text{aq}}}{dt}: f^s_{8,1} = \frac{1}{u_{h_{\text{aq}}}} u_{k_{\text{off}}} k_{\text{off}} u_{h_{\text{aq}}} h_{\text{aq}}, \quad f^s_{8,2} = \frac{1}{u_{h_{\text{aq}}}} u_{k_{\text{on}}} k_{\text{on}} r_{\text{aq}} u_{c_{\text{aq}}} c_{\text{aq}}, \quad f^s_{8,3} = \frac{1}{u_{h_{\text{aq}}}} \frac{u_{V_{\text{vit}}} V_{\text{vit}}}{u_{V_{\text{aq}}} V_{\text{aq}}} u_{k^h_{\text{el}}} k^h_{\text{el}} u_{h_{\text{vit}}} h_{\text{vit}},$$
$$f^s_{8,4} = \frac{1}{u_{h_{\text{aq}}}} \frac{u_{CL} CL}{u_{V_{\text{aq}}} V_{\text{aq}}} u_{h_{\text{aq}}} h_{\text{aq}}.$$

We then equate the scaled functions with the pre-scaled functions ($f_{i,k} = f^s_{i,k}$), finding

$$v_{\text{vit}} = r_{\text{vit}} = c_{\text{vit}} = h_{\text{vit}} = c_{\text{aq}} = h_{\text{aq}} = 1, \quad \text{and}$$
$$k_{\text{off}} = k_{\text{on}} = V_{\text{in}} = V_{\text{vit}} = k^v_{\text{el}} = k^r_{\text{el}} = k^c_{\text{el}} = k^h_{\text{el}} = V_{\text{aq}} = CL = 1,$$

thus providing the conclusion that the system described in Supplementary Material S1 is structurally identifiable, to within the limits of the Castro and de Boer[4] algorithm.

S3. Sensitivity analysis over time

The sensitivity analysis across time for each molecular species for which there is currently no data (v_{vit}, r_{vit}, c_{vit}, h_{vit}, c_{aq} and h_{aq}) are shown in Figure S3.1. The first-order sensitivities are shown by the solid lines and the total-order sensitivities are shown by the dashed lines. The color scheme for each subplot is shown in Figure S3.1B.

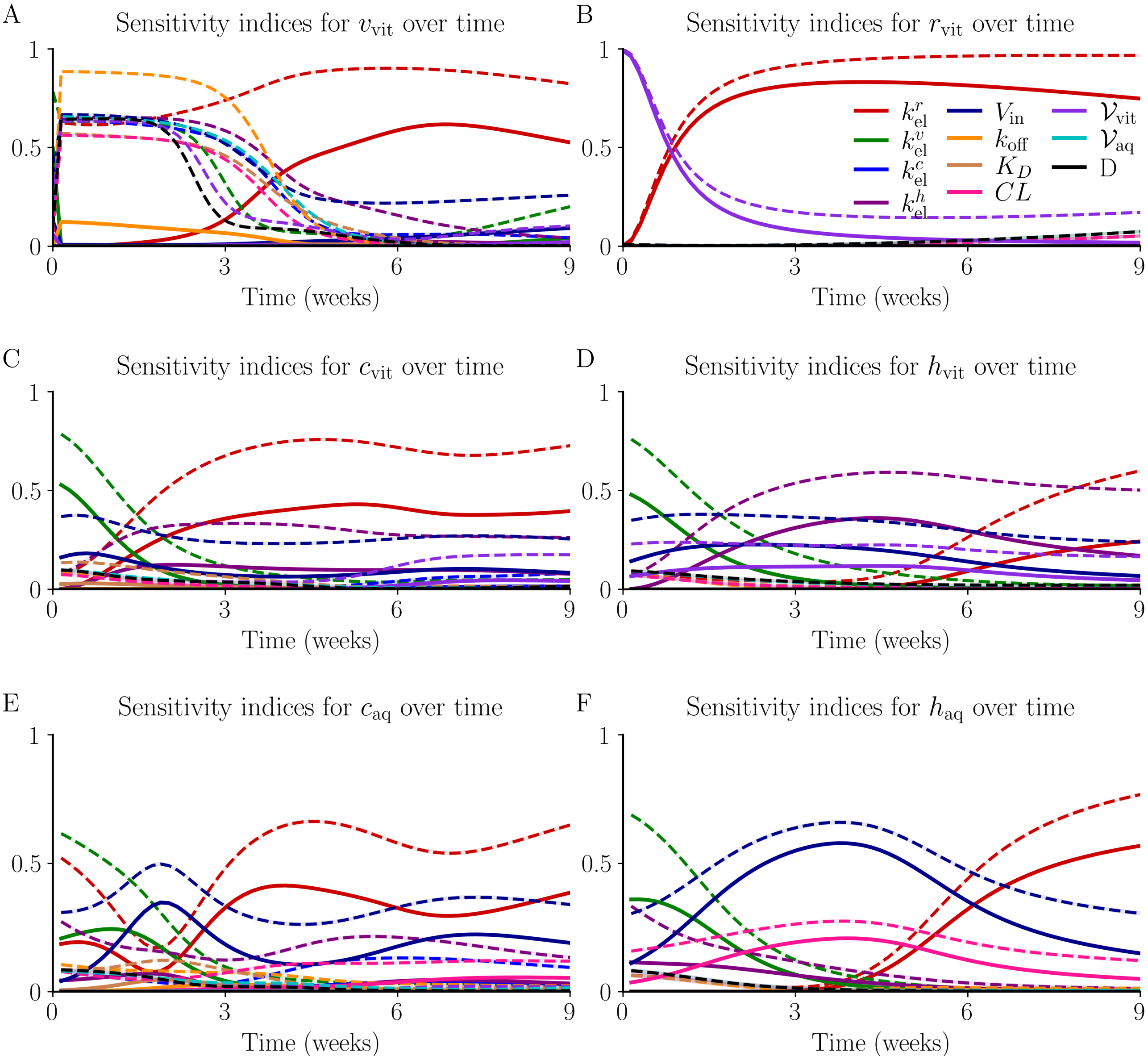

Figure S3.1: Sensitivity indices for each parameter for A v_{vit}, B r_{vit}, C c_{vit}, D h_{vit}, E c_{aq}, and F h_{aq} over time. The first-order sensitivities are shown by the solid lines, and the total-order sensitivities are shown by the dashed lines. The 95% confidence interval is given by the very narrow shaded region around each curve, however is too narrow to be distinguished from the curve. The color scheme for each subplot is shown in B.

S4. Prior selection

Supplementary Table S4.1 shows the prior mean±SD, bounds and units for each parameter. A plot of each prior distribution is provided in the rightmost column.

S5. Time-series pharmacokinetic data extracted from Niwa *et al.*[5]

The averaged time-series pharmacokinetic data extracted from the aqueous humor of three healthy cynomolgus macaques published by Niwa *et al.*[5] are shown in Table S5.1. Data were extracted using WebPlotDigitizer (version 4.5). Data below the lower limit of quantification (LLQ) (9 pg/ml and 0.156 ng/ml for aqueous humor VEGF and ranibizumab, respectively) are denoted by an asterisk (*).

Table S4.1: The prior distributions for each model parameter were constructed with reference to the literature values given in Table 1 and designed to provide weakly informative priors, corresponding to truncated normals. The SD bars in the Niwa *et al.*[5] data set were used to create the priors σ_1 and σ_2, respectively.

$P(\theta_i)$	Mean ± SD	Bounds	Units	Prior distribution
k_{el}^{r}	0.24 ± 0.48	$[0,1]$	day^{-1}	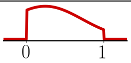
k_{el}^{v}	0.37 ± 0.74	$[0,1]$	day^{-1}	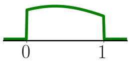
k_{el}^{c}	0.21 ± 0.42	$[0,1]$	day^{-1}	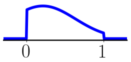
k_{el}^{h}	0.18 ± 0.36	$[0,1]$	day^{-1}	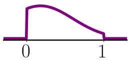
V_{in}	4.20 ± 8.40	$[0,20]$	pM·mL·day^{-1}	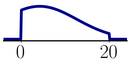
k_{off}	1.30 ± 1.00	$[0,10]$	day^{-1}	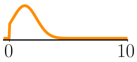
K_D	1500 ± 10^5	$[0,10^8]$	pM	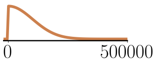
CL	2.86 ± 1.00	$[0,10]$	mL·day^{-1}	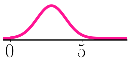
σ_1	0.24 ± 0.25	$[0,10]$	pM	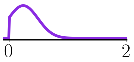
σ_2	0.15 ± 0.25	$[0,10]$	pM	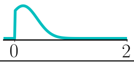

Table S5.1: The averaged time-series pharmacokinetic data obtained from the aqueous humor of three healthy cynomolgus macaques published by Niwa *et al.*[5]. Data below the lower limit of quantification (LLQ) (9 pg/ml and 0.156 ng/ml for aqueous humor VEGF and ranibizumab, respectively) are indicated with a superscript asterisk (*).

Time (days)	Aqueous humor VEGF (pM)	Aqueous humor ranibizumab (pM)
0	99.700	—
1	0.000*	51300.0
3	0.051*	19512.2
7	0.083*	8448.3
14	0.114*	1396.1
21	0.146*	131.0
28	65.117	9.0
35	77.649	3.2
42	98.412	0.3
49	98.139	0.1*
56	100.000	—

S6. Consideration of data density and multiplicative noise on data correction technique choice

To consider how the quality of a given data set should alter the choice of data correction technique chosen to handle data below the LLQ, we conducted an additional analysis using a series of synthetic data sets of varying quality. Each synthetic data set was generated with specific levels of noise ($\sigma_N = 0.1$, 0.25 or 0.5) and data density (5, 10 or 20 data points) using a reference parameter set $\boldsymbol{\theta}_{\text{ref}}$, which may be deduced from the vertical black lines in Supplementary Figure S6.1. To remove biases introduced by random perturbations during data generation, we generated ten data sets for each combination of noise level and data density. For each data set, we applied the M5, M6 and M7 data correction techniques, conducted a Bayesian analysis, and then compared the inferred posterior distributions, as shown in Figure S6.1. Figure S6.1 shows the normalised average posterior distributions for each posterior distribution at each level of noise and data density with each data correction technique (color corresponding to the legend in the top right of Figure S6.1F). Excluding the maximum noise at the maximum data density considered ($\sigma_N = 0.5$ with 20 data points), M7 (green) provided the posterior distributions that were most closely aligned with the posterior distributions inferred from the uncorrected the data set (grey shaded distribution with a black outline) for most parameters. The posterior distributions inferred from the uncorrected synthetic data serve as the gold standard, as the data remain unaltered and are not affected by limits of quantification. When we have high data density (≥ 20 data points) and high noise ($\sigma_N = 0.5$), removing the data below the LLQ (M6; red) appears to be the optimal approach, as is clearly evident for k_{el}^r, k_{el}^v, k_{el}^c, k_{el}^h and k_{off} (Figure S6.1A–F). Regardless of data quality, we consistently observe that the data correction technique M5 (blue) tends to overestimate k_{el}^r (Figure S6.1A) while underestimating k_{el}^v, k_{el}^c, k_{el}^h and k_{off} (Figure S6.1B–F). Furthermore, the posterior distributions of k_{off} (Figure S6.1F) are increasingly weighted towards zero and away from the input parameter as the data density increases for the M5 data correction technique. This is likely because the number of data points falling below the LLQ is increased, leading to a greater number of data points being overestimated by this data correction technique.

These findings comparing data correction techniques M5, M6 and M7 (Figure S6.1) indicate that, for this model, the implementation of the M7 data correction technique is the most suitable choice. However, in data sets containing high levels of noise and collected at a high density, the M6 data correction technique appears to be the most appropriate for this model.

S7. Comparison of data correction techniques applied to the Niwa *et al.*[5] data set

The posterior distributions following data correction techniques M5, M6 and M7 applied to the Niwa *et al.*[5] data set are shown in Figure S7.1. Niwa *et al.*[5] used data correction technique M7 (third row). We do not have accurate data for the values below the LLQ for the Niwa *et al.*[5] data set; as such we are unable to analyse these data without a data correction technique.

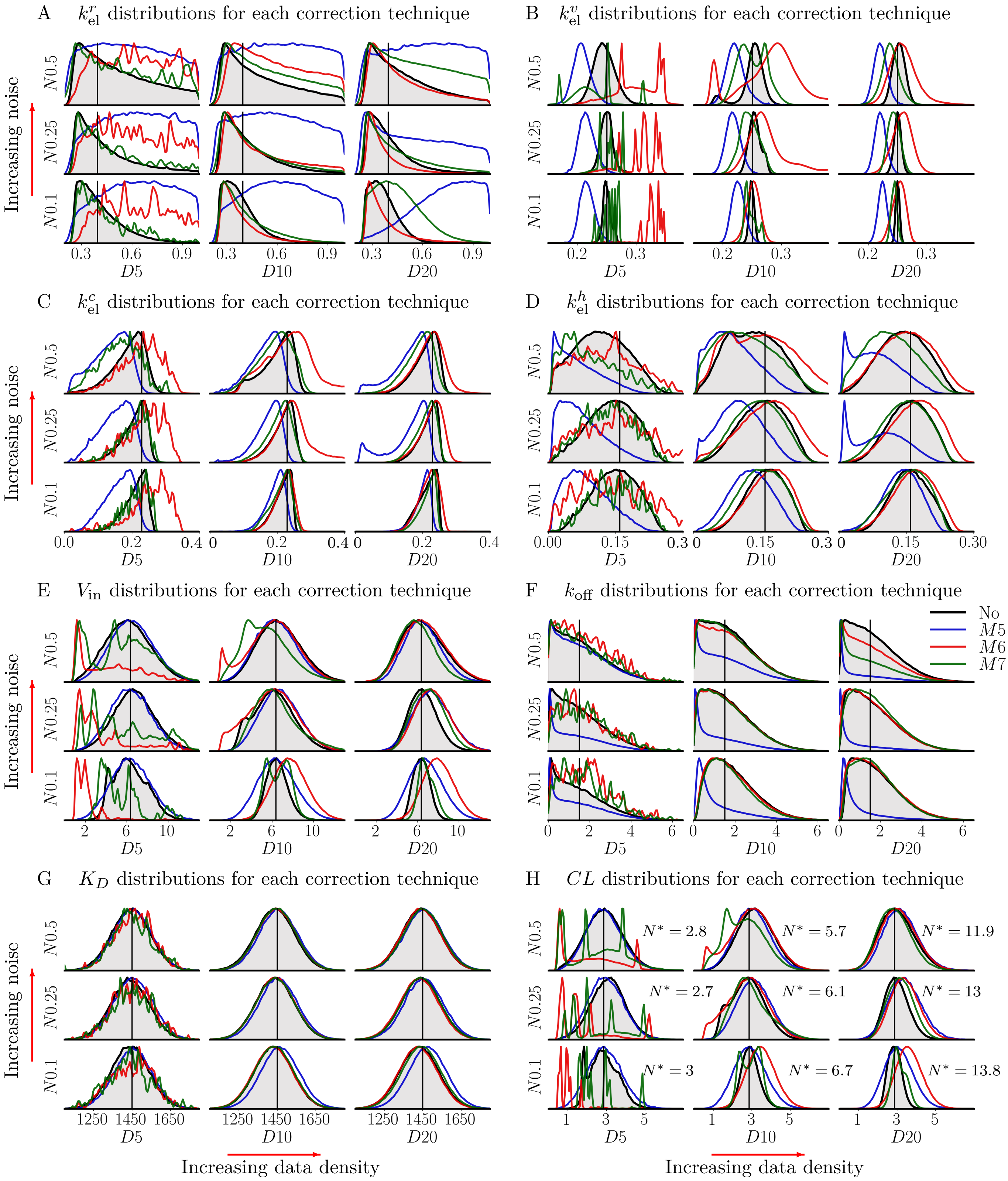

Figure S6.1: Normalised average inferred posterior distributions for synthetic data sets with varying data density (5, 10 and 20; increasing in the horizontal direction) and noise ($\sigma_N = 0.1$, 0.25 and 0.5; increasing in the vertical direction). $N0.1$ corresponds to $\sigma_N = 0.1$, and analogously for other values of σ_N, with $D5$ corresponding to 5 data points for instance. Each data set has either no correction implemented (grey with black outline), or the data correction techniques M5 (blue), M6 (red) or M7 (green), while the vertical black lines represent the reference parameter values. The N^* values in subplot H represent the average number of data points below the LLQ. The color scheme for all subplots is shown in subplot F.

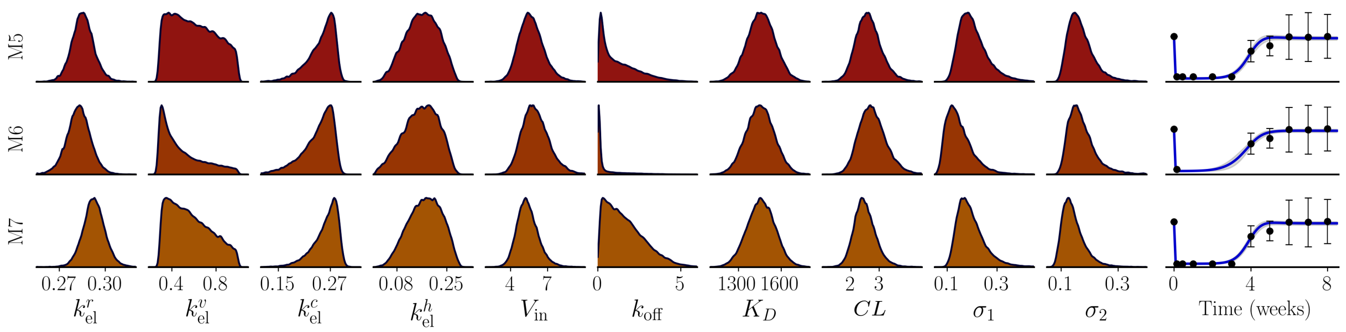

Figure S7.1: Inferred posterior distributions obtained from the Niwa *et al.*[5] data set following data correction techniques M5 (first row), M6 (second row) and M7 (third row). Each distribution has been normalised by the maximum of the distribution. The model fits following each correction technique are shown in the rightmost column.

S8. Posterior distributions with DEMCMC chains

Figures S8.1 and S8.2 show each posterior distribution (left) along with the corresponding prior distribution and the DEMCMC trace (right) for the base model described and the adapted model, respectively, where the adapted model allows for the possibility that a proportion, γ, of partially bound VEGF and a proportion, η, of fully bound VEGF are measured by the ELISA technique. The posterior distributions are shown in the left column of Figures S8.1. The left column of Figures S8.1 and S8.2 reproduces the posterior distributions shown along the diagonals of Figure 4 and Figure 7A. Replotting the posterior distributions alongside the prior distributions for each parameter clearly shows that each posterior distribution is more narrow and entirely contained within the corresponding prior distribution, thus showing that we have identifiability for each model parameter and the prior distributions did not present any undue restraints on the posterior distributions. We also see that the chains (right column) in Figures S8.1 and S8.2 have converged before the 5000 iterations.

Figure S8.3 provides a direct comparison between the posterior distributions inferred for the base model and for the extended model, showing that there is little variation in the posterior distributions between the two models, with the exception of K_D, $k^v_{\rm el}$ and σ_1. The K_D posterior distribution has a wider spread in the extended model, indicating increased uncertainty around this parameter. The posterior distribution for $k^v_{\rm el}$ is skewed left, slowing the transfer of VEGF from the vitreous to the aqueous, possibly compensating for the additional contribution of VR and RVR in the aqueous humor free VEGF measurements. Finally, the σ_1 posterior distribution has a wider spread in the adapted model, suggesting that the adapted model has a poorer fit compared to the base model. Figure S8.4 shows the model fits for the base model (blue) and the adapted model (red) overlaid. Again we observe that the adapted model provides an inferior fit for the VEGF aqueous measurement between weeks $1-6$, around the beginning of the transition region (P3).

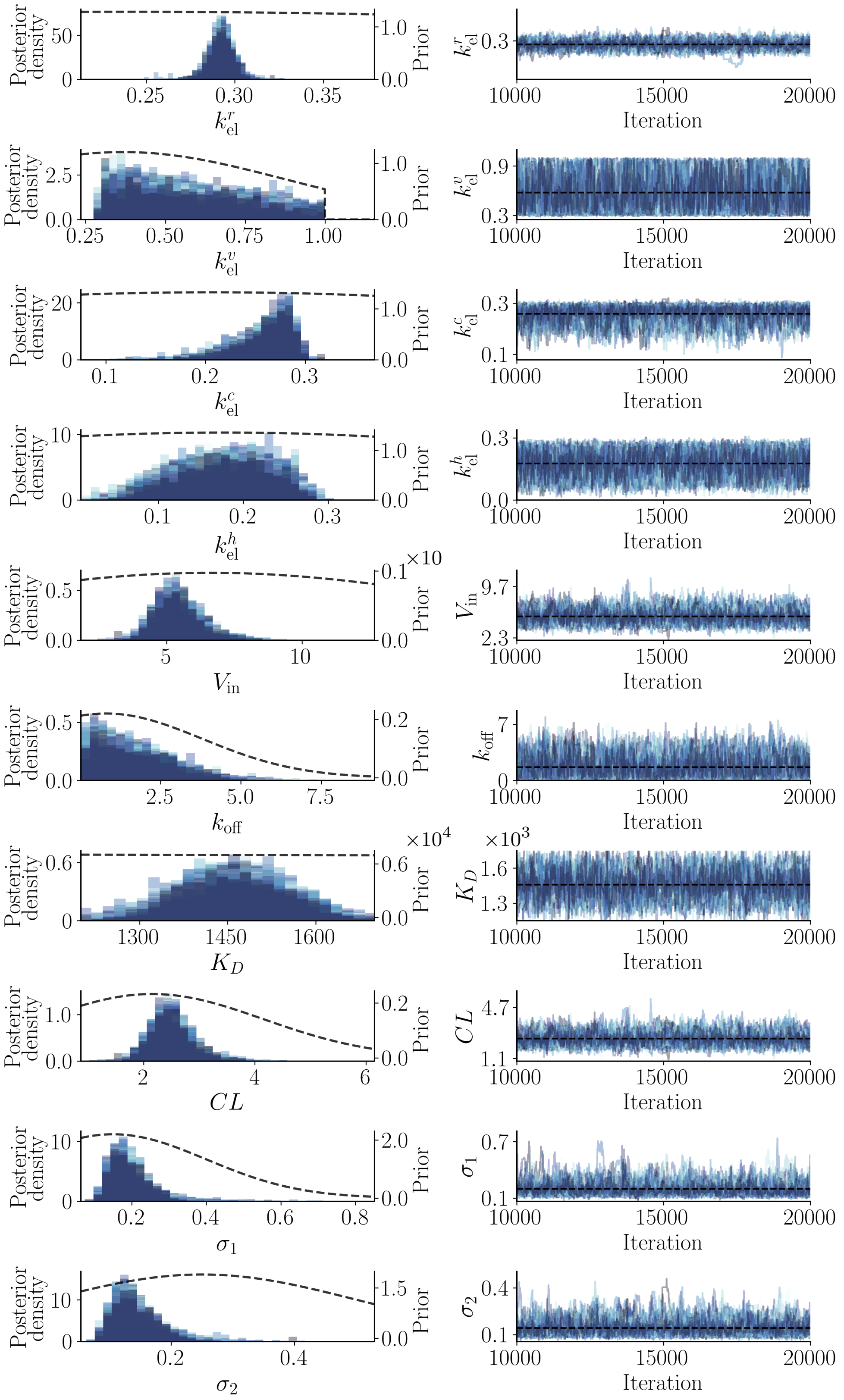

Figure S8.1: The inferred posterior distributions (left) alongside the DEMCMC trace (right) for each parameter for the semi-mechanistic two-compartment model. The prior distributions (black dashed) are shown via the right axis of the plots in the first column for comparison with the posterior distributions.

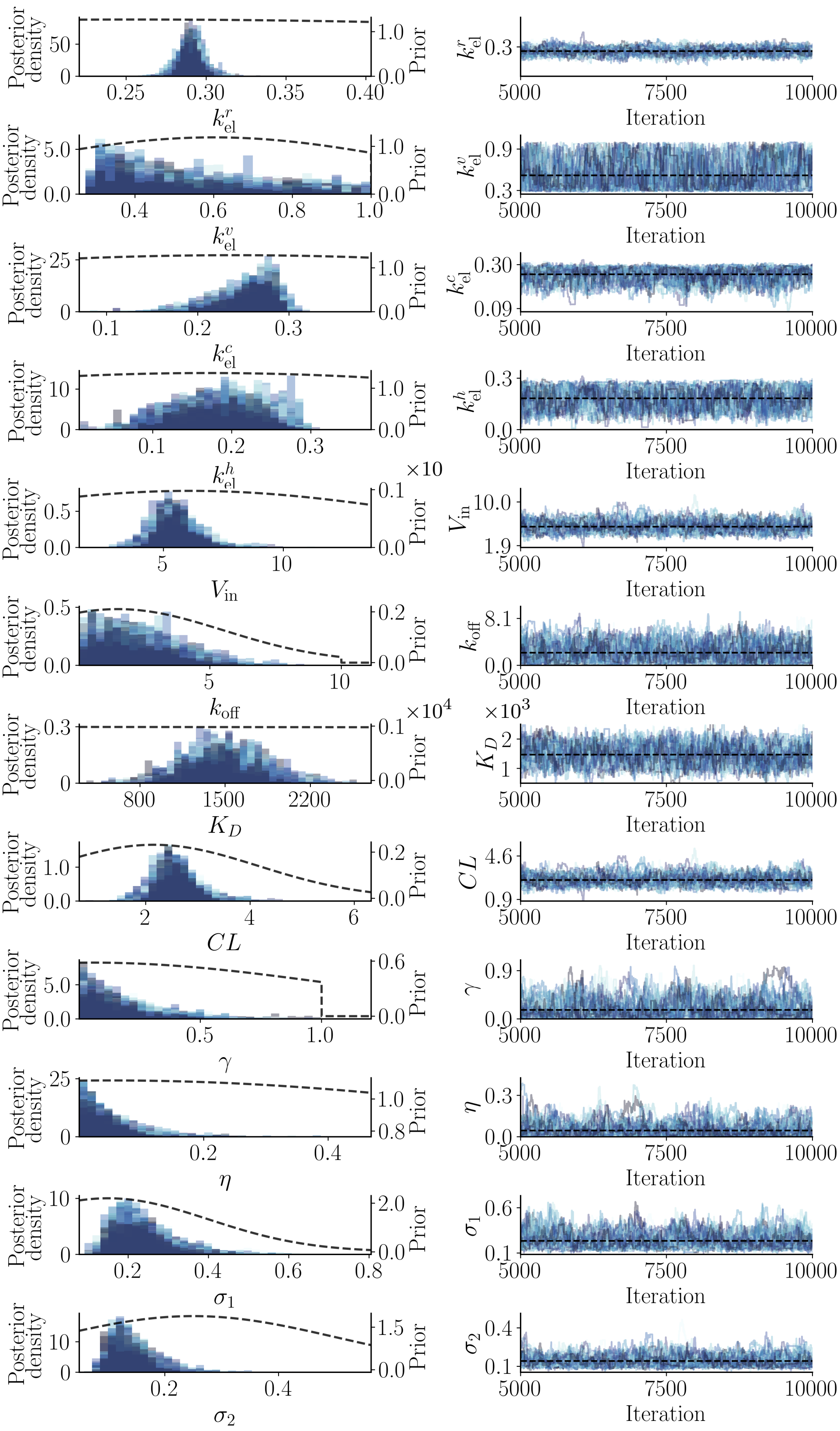

Figure S8.2: The inferred posterior distributions (left) alongside the DEMCMC trace (right) for each parameter for the adapted model where the parameters γ and η have been introduced to describe the proportion of partially and fully bound VEGF captured by the ELISA measurements, respectively. The prior distributions (black dashed) are shown via the right axis of the plots in the first column for comparison with the posterior distributions.

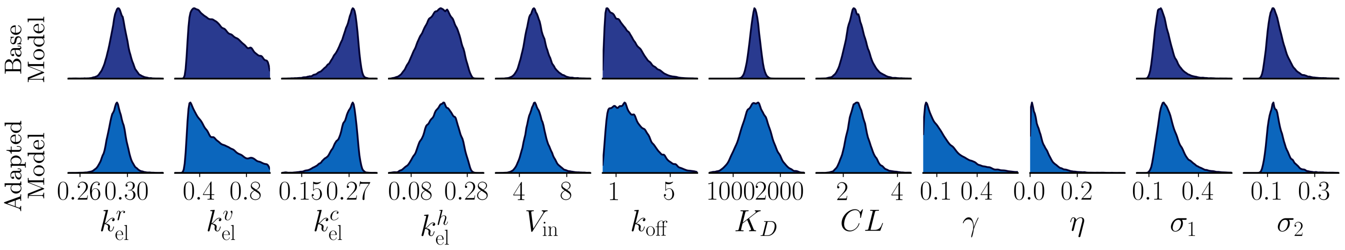

Figure S8.3: A comparison of the posterior distributions inferred from the Niwa *et al.*[5] data set: (1) using the model described by Supplementary Equations (S1.1) (Base model, top row), or (2) using the model described by Supplementary Equations (S1.1) and assuming that the measured aqueous VEGF contains some proportion of partially (γ) and fully (η) bound VEGF (Equation (4)) (Adapted model, bottom row).

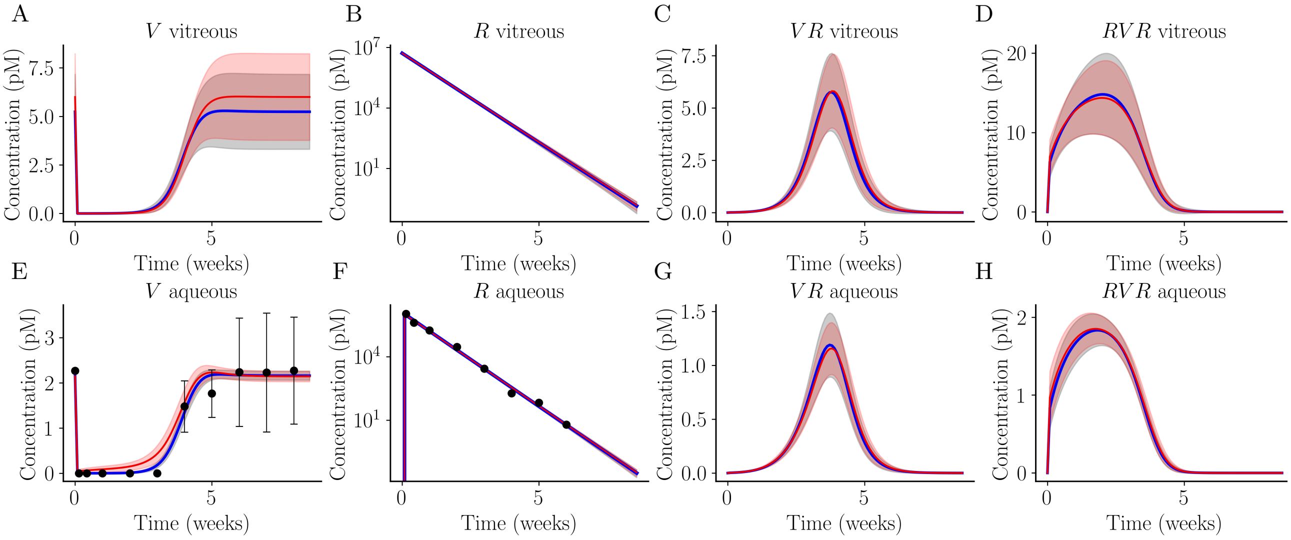

Figure S8.4: A comparison of two semi-mechanistic two-compartment models of intraocular ranibizumab and VEGF PK/PD following a single IVT bolus of ranibizumab. Model 1 (blue) assumes that the putative capture antibody of the Quantikine VEGF ELISA measures only free VEGF (base model). Model 2 (red) assumes that the putative capture antibody of the Quantikine VEGF ELISA also captures some proportion of partially and fully bound VEGF (adapted model). The mean model fits are shown by the solid line with the shaded regions showing a single SD around the mean model fits. From left to right, the top row shows the vitreous concentration of (A) VEGF (V), (B) ranibizumab (R), (C) partially bound VEGF (VR) and (D) fully bound VEGF (RVR). Similarly, the bottom row (E–H) shows the aqueous concentration of each molecular species in the same order. The mean±SD of the aqueous VEGF and the aqueous ranibizumab data extracted from Niwa *et al.*[5] are shown by the black circles and error bars in subplots E and F.

Bibliography

[1] L. A. Hutton-Smith, E. A. Gaffney, H. M. Byrne, P. K. Maini, D. Schwab, and N. A. Mazer, "A mechanistic model of the intravitreal pharmacokinetics of large molecules and the pharmacodynamic suppression of ocular vascular endothelial growth factor levels by ranibizumab in patients with neovascular age-related macular degeneration," *Molecular Pharmaceutics*, vol. 13, no. 9, pp. 2941–2950, 2016.

[2] J. Penn, A. Madan, R. B. Caldwell, M. Bartoli, R. Caldwell, and M. Hartnett, "Vascular endothelial growth factor in eye disease," *Progress in Retinal and Eye Research*, vol. 27, no. 4, pp. 331–371, 2008.

[3] N. Ferrara, L. Damico, N. Shams, H. Lowman, and R. Kim, "Development of ranibizumab, an anti–vascular endothelial growth factor antigen binding fragment, as therapy for neovascular age-related macular degeneration," *Retina*, vol. 26, no. 8, pp. 859–870, 2006.

[4] M. Castro and R. J. de Boer, "Testing structural identifiability by a simple scaling method," *PLOS Computational Biology*, vol. 16, no. 11, p. e1008248, 2020.

[5] Y. Niwa, M. Kakinoki, T. Sawada, X. Wang, and M. Ohji, "Ranibizumab and aflibercept: intraocular pharmacokinetics and their effects on aqueous VEGF level in vitrectomized and nonvitrectomized macaque eyes," *Investigative Ophthalmology & Visual Science*, vol. 56, no. 11, pp. 6501–6505, 2015.
